# Malignant Transformation Involving *CXXC4* Mutations Identified in a Leukemic Progression Model of Severe Congenital Neutropenia

**DOI:** 10.1016/j.xcrm.2020.100074

**Published:** 2020-08-25

**Authors:** Patricia A. Olofsen, Szabolcs Fatrai, Paulina M.H. van Strien, Julia C. Obenauer, Hans W.J. de Looper, Remco M. Hoogenboezem, Claudia A.J. Erpelinck-Verschueren, Michael P.W.M. Vermeulen, Onno Roovers, Torsten Haferlach, Joop H. Jansen, Mehrnaz Ghazvini, Eric M.J. Bindels, Rebekka K. Schneider, Emma M. de Pater, Ivo P. Touw

**Affiliations:** 1 Department of Hematology, Erasmus University Medical Center, Rotterdam 3015 CN, the Netherlands; 2 Munich Leukemia Laboratory (MLL), Munich 81377, Germany; 3 Department of Laboratory Medicine, Radboud University Medical Center, Nijmegen 6525 GA, the Netherlands; 4 Department of Developmental Biology, iPS Core Facility, Erasmus University Medical Center, Rotterdam 3015 CN, the Netherlands

**Keywords:** severe congenital neutropenia, AML, CSF3R, RUNX1, CXXC4, TET2, pro-inflammatory signaling, growth factor therapy, leukemia predisposition

## Abstract

Severe congenital neutropenia (SCN) patients treated with CSF3/G-CSF to alleviate neutropenia frequently develop acute myeloid leukemia (AML). A common pattern of leukemic transformation involves the appearance of hematopoietic clones with CSF3 receptor (*CSF3R*) mutations in the neutropenic phase, followed by mutations in *RUNX1* before AML becomes overt. To investigate how the combination of CSF3 therapy and *CSF3R* and *RUNX1* mutations contributes to AML development, we make use of mouse models, SCN-derived induced pluripotent stem cells (iPSCs), and SCN and SCN-AML patient samples. CSF3 provokes a hyper-proliferative state in *CSF3R*/*RUNX1* mutant hematopoietic progenitors but does not cause overt AML. Intriguingly, an additional acquired driver mutation in *Cxxc4* causes elevated CXXC4 and reduced TET2 protein levels in murine AML samples. Expression of multiple pro-inflammatory pathways is elevated in mouse AML and human SCN-AML, suggesting that inflammation driven by downregulation of TET2 activity is a critical step in the malignant transformation of SCN.

## Highlights

Combinatorial *CSF3R* and *RUNX1* mutations seen in SCN-AML do not result in AML in mice
An additional mutation in *Cxxc4* causes AML development in *CSF3R/RUNX1* mutant mice
Mutant CXXC4 protein is more stable than wild-type and reduces TET2 protein levels
*CXXC4* mutations are also found in *de novo* AML patients

## Graphical Abstract

**Figure:**
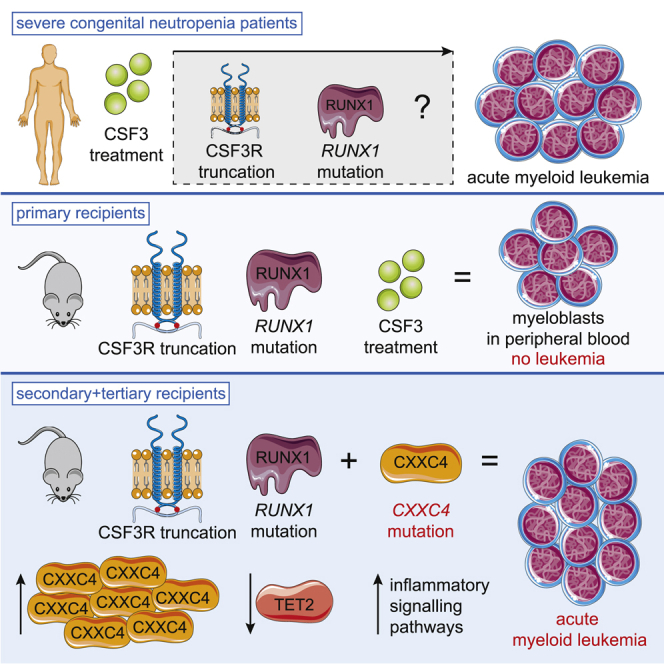

## Teaser

Olofsen et al. show that acquisition of a mutation in *Cxxc4* results in increased CXXC4 protein levels, reduced TET2 protein, increased inflammatory signaling, and leukemic progression of a *CSF3R/RUNX1* mutant mouse model of severe congenital neutropenia (SCN).

## Introduction

Severe congenital neutropenia (SCN) is an inherited bone marrow failure syndrome characterized by an almost complete lack of neutrophils, leading to life-threatening bacterial infections.^1^ SCN is most often caused by autosomal dominant mutations in *ELANE*, the gene encoding neutrophil elastase, but how these mutations give rise to severe neutropenia is still largely unknown.^1^ In the majority of SCN patients, neutropenia is successfully alleviated by life-long administration of colony-stimulating factor 3 (CSF3), also known as granulocyte CSF (G-CSF).^2^ SCN patients are at risk of developing high-risk myelodysplastic syndrome (MDS) or acute myeloid leukemia (AML), with a reported median incidence of 21%, 15 years after initiation of CSF3 treatment.^3^^,^^4^ Leukemic progression correlates with the appearance of hematopoietic clones with somatic mutations in *CSF3R*, resulting in a truncated form of CSF3R with defective internalization and aberrant signaling properties.^5^ These mutant clones arise before MDS or AML becomes clinically overt, indicating that additional defects are needed for malignant transformation.

Mutations in *RUNX1* are the most prevalent mutations (64.5%) acquired during leukemic transformation of SCN and typically occur in clones already harboring somatic *CSF3R* mutations (85%).^6^ RUNX1 is a member of the runt transcription factor family and is essential for fetal hematopoiesis.^7^^,^^8^ RUNX proteins share the highly conserved runt homology domain (RHD) involved in DNA binding and in the interaction with the regulatory protein core-binding factor β.^9^ In SCN-MDS/AML, mainly *RUNX1* missense mutations in the RHD were found in combination with the *CSF3R*-truncating mutations.^6^^,^^10^ Although recurrent mutations in other genes (e.g., encoding the epigenetic modifiers *ASXL1* and *SUZ12*) were also found, these were far less frequent. How truncated *CSF3R* mutants and *RUNX1* mutations in conjunction with disease-causing *ELANE* mutations contribute to AML development in SCN is unknown. To address this question, we used a combination of *in vivo* mouse models and *in vitro* patient-derived induced pluripotent stem cell (iPSC) models engineered to express patient-specific *CSF3R* and *RUNX1* mutations.

## Results

### *CSF3R* and *RUNX1* Mutations Elevate CSF3-Induced Proliferation of LK Progenitor Cells

**Figure 1:**
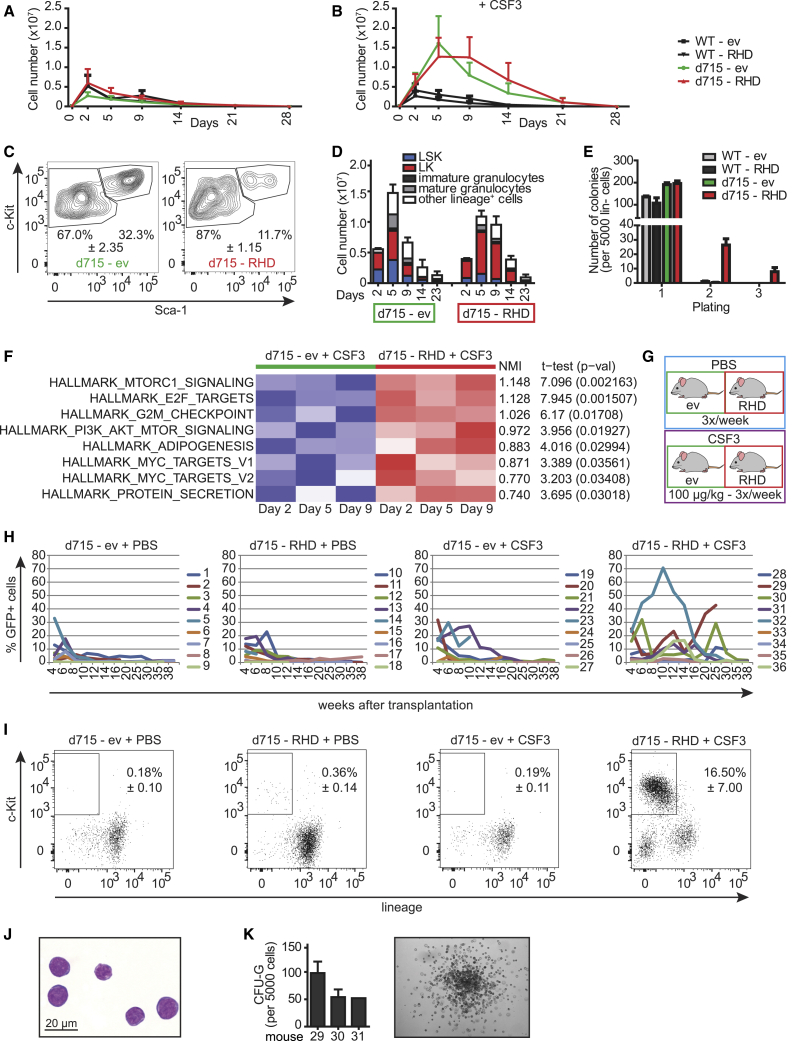
*Csf3r*-d715 and *RUNX1*-RHD in Conjunction with CSF3 Treatment Causes the Expansion of LK Cells (A–B) Proliferation of empty vector (ev)- or *RUNX1*-RHD virus-transduced cells in stem cell expansion medium (A) without CSF3 and (B) with 50 ng/mL CSF3. Error bars show one-sided standard error of the mean (SEM) of 3 independent experiments (biological replicates). (C) Representative FACS contour plots showing LK/LSK distribution of d715-ev (left panel) and d715-RHD (right panel) in CSF3-supplemented cultures (day 9), with ± indicating SEM. (D). Distribution of immature and differentiated cell types in CSF3-containing cultures spanning 23 days. Error bars show one-sided SEM of 3 independent experiments (biological replicates). (E). CSF3-induced colony formation in primary colony assays and in secondary and tertiary replating cultures (n = 3 independent experiments [biological replicates] performed in triplicate [technical replicates]). (F). Comparative transcriptome profiles of CSF3-stimulated d715-ev and d715-RHD LK cells on days 2, 5, and 9 of culture (n = 1 sample per time point). (G). Experimental setup. CSF3/PBS administration started 4 weeks after transplantation. (H). Longitudinal analysis of GFP-expressing cells in PB (n = 9 mice per experimental group, biological replicates). (I). Accumulation of GFP-expressing LK cells in PB of CSF3-treated d715-RHD-transplanted mice but not in other groups. Data are from PB samples taken 14 and 16 weeks after transplantation (n = 11 or 12 per group), with ± indicating SEM. (J) Myeloblast morphology of FACS-purified PB LK cells. The scale bar indicates 20 μm. (K) CSF3-induced colony formation of PB LK cells (mice 29, 30, and 31) and representative example of the colony (n = 1 experiment in triplicate [technical replicates]). See also Figure S1.

We first investigated how a *RUNX1* mutation affects CSF3 responses of mouse hematopoietic stem and progenitor cells (HSPCs) *in vitro*. The patient-specific truncated CSF3R, *Csf3r*-d715, or wild-type (WT) lineage-depleted bone marrow (BM) cells expressing the patient-specific *RUNX1*-D171N (RHD) mutation (Figures S1A and S1B) or an empty vector (ev) control were cultured in stem cell expansion medium with or without CSF3 (Figures 1A and 1B). In the absence of CSF3, no differences in absolute cell numbers between any of the conditions were seen (Figure 1A), whereas addition of CSF3 induced a strong and sustained hyperproliferative response in *Csf3r*-d715 cells that was slightly, but not significantly, extended by mutant RUNX1 (Figure 1B). *Csf3r*-d715 cells expressing RUNX1-RHD (d715-RHD cells) cultured in CSF3 medium showed selective expansion of lineage-negative, Sca1-negative, c-Kit-expressing (LK) cells relative to d715-ev control cells, which showed a more equal distribution between LK and more primitive LSK (Lineage^−^, Sca1^+^, c-Kit^+^) cells (Figure 1C). Notably, d715-RHD cell cultures consistently showed a higher number of LK cells and reduced numbers of differentiated cells relative to the ev controls from day 5 onward (Figure 1D). d715-RHD cells also displayed a moderate ability of secondary and tertiary replating in CSF3-containing colony cultures, whereas d715-ev or WT-RHD cells lacked this capability (Figure 1E). Comparative transcriptome analysis and subsequent single-sample gene set enrichment analysis (ssGSEA) of LK cells from CSF3-supplemented cultures purified by fluorescence-activated cell sorting (FACS) on days 2, 5, and 9 showed higher activation of hallmark pathways of cell proliferation (E2F, G2M checkpoint, and MYC) and metabolism (mTORC1) in d715-RHD cells relative to d715-ev cells (Figure 1F). In summary, these results show that mutations in *Csf3r* and *RUNX1* have additive effects on proliferation of myeloid cells, leading to expansion of LK cells and reduced production of more mature myeloid cells.

### *In Vivo* Expansion of *Csf3r*-d715/*RUNX1*-RHD Mutant HSPCs Induced by Sustained CSF3 Administration

**Figure 2:**
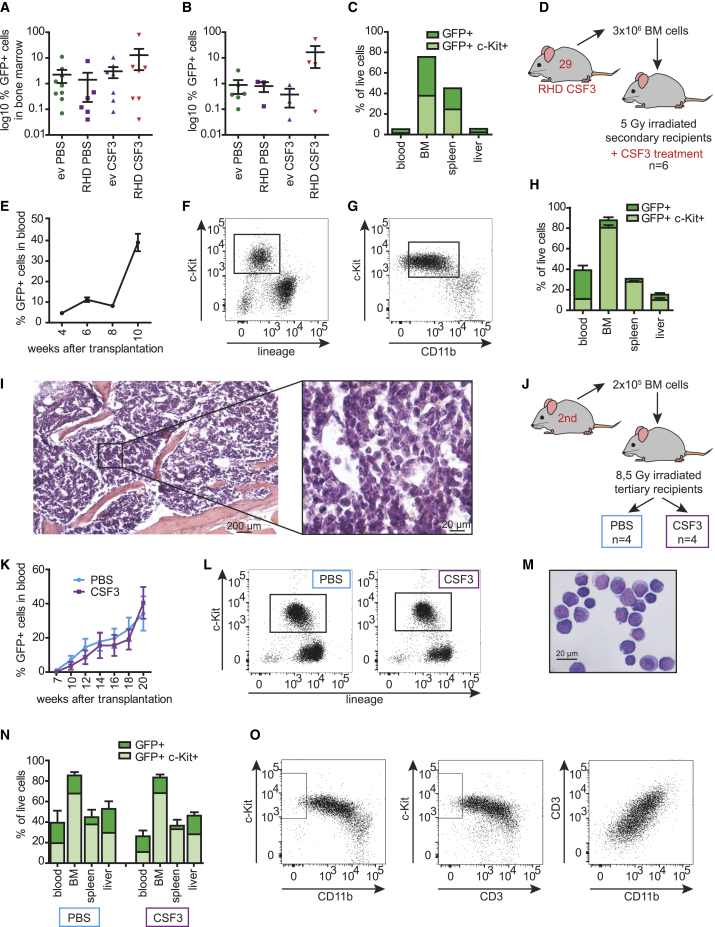
Spleen Infiltration and CSF3-Independent Leukemogenic Properties of d715-RHD Cells (A and B) Distribution of GFP-expressing cells in (A) BM and (B) spleen in primary recipients, showing enhanced spleen infiltration in CSF3-treated d175-RHD mice. (C). Relative distributions of total GFP^+^ and GFP^+^c-Kit^+^ cells in various organs of mouse 29. (D) Secondary transplant setup; cells from mouse 29 were transplanted into 6 recipients. (E–G) Expansion of GFP^+^ cells in PB of 6 secondary recipients (E, biological replicates), with (F) the c-Kit^+^ immunophenotype and (G) intermediate CD11b expression on c-Kit^+^ cells. (H) Organ distribution of GFP^+^ and GFP^+^c-Kit^+^ cells. (I) H&E staining of the spine, showing a hypercellular BM with more than 90% myeloblast infiltration. The scale bars indicate 200 and 20 μm, respectively. (J) Tertiary transplantation setup. (K) Equal expansion of GFP^+^ cells in PB of CSF3-treated (n = 4) or PBS-treated (n = 4) mice (biological replicates). (L) The c-Kit^+^ phenotype of PB GFP^+^ cells in CSF3- and PBS-treated mice. (M) Blast morphology of FACS-purified c-Kit^+^ PB cells. (N) Organ distribution of GFP^+^ and GFP^+^c-Kit^+^ cells in PBS- or CSF3-treated mice (n = 4 per group). (O) FACS analysis showing an immature (c-Kit^+^) mixed myeloid (CD11b^+^) and T cell (CD3^+^) immunophenotype of AML blasts; the boxes indicate c-Kit^+^CD11b^−^CD3^−^. Error bars represent SEM. See also Figure S2.

To address how the *Csf3r* and *RUNX1* mutations in combination with CSF3 administration affect hematopoiesis *in vivo*, we performed transplantation experiments using lentivirally transduced lineage-negative *Csfr*-d715 BM cells (Figure S1C). Western blot analysis confirmed the presence of human RUNX1-D171N protein (Figure S1D). Transduction efficiency, analyzed by flow cytometry based on the presence of the IRES-GFP cassette in the lentiviral vector (Figure S1C), was approximately 40%–50% in lineage-negative BM cells (Figure S1E) and 65% in d715-RHD or d715-ev LSK cells (Figure S1F). These cells were transplanted into lethally irradiated WT recipients. Starting 4 weeks after transplantation, mice were treated three times per week with CSF3 or PBS (n = 9 per group; Figure 1G). GFP^+^ cells in the peripheral blood (PB) within each group varied between mice (Figure 1H). Overall, mice transplanted with d715-RHD BM cells and treated with CSF3 showed the highest level of long-term chimerism; i.e., exceeding 16 weeks following transplantation and 12 weeks after initiation of CSF3 administration (Figure 1H). Fluctuations in GFP^+^ PB cell percentages in individual mice over time suggest that CSF3 propagated the persistence rather than a dominant outgrowth of d715-RHD clones. Strikingly, CSF3-treated d715-RHD mice had significant percentages (16.5% average, SEM 7%) of GFP^+^ LK cells in PB, which was not seen in the other experimental groups (Figure 1I; <1%). These cells were characterized as myeloblasts by morphology (Figure 1J) and comprised 1%–2% myeloid colony-forming cells (Figure 1K; 50–100 colonies/5,000 cells), equivalent to the observations of *in vitro* cultures (Figures 1C–1E). Although more than 5% myeloblasts in the PB are a characteristic of AML, none of the CSF3-treated d715-RHD mice showed acute symptoms of AML, such as bleeding or anemia. In addition, no defect in myeloid differentiation was observed as percentages of GFP^+^CD11b^+^GR1^+^ neutrophils in PB were comparable between groups (Figure S1G). At the time of sacrifice, no excessive GFP^+^ blasts in the BM were seen, with the exception of mouse 29 (Figure 2A). On the other hand, increased numbers of GFP^+^ cells were detected in the spleens of d715-RHD CSF3-treated mice (Figure 2B). These findings suggest that the CSF3-treated d715-RHD mice display a pre-malignant phenotype characterized by cellular but not symptomatic features of overt AML.

### Premalignant *Csf3r/RUNX1* Mutant BM Cells Progress to AML in Secondary Recipients

The ability to engraft in secondary recipients is a hallmark of murine leukemia models. To determine to what extent this applied to the pre-malignant nature of d715-RHD BM cells from CSF3-treated mice, we transplanted BM cells from mouse 29, which presented with the highest level of engraftment 25 weeks after primary transplantation, in sub-lethally irradiated secondary recipients that were subsequently treated with CSF3 (n = 6; Figures 2C and 2D). GFP^+^c-Kit^+^ cells were detectable in the blood 4 weeks after transplantation and could be detected until the end of the experiment (Figures 2E and 2F). Contrary to the LK cells expanding in primary recipients, these cells weakly co-expressed the myeloid marker CD11b, suggestive of a myeloid bias within the LK compartment (Figure 2G). High percentages of GFP^+^c-Kit^+^ cells were present in the BM, spleen, and liver (Figure 2H). Prior to sacrifice, mice were severely anemic because of an erythroid differentiation block at the CD71^+^Ter119^+^ basophilic and early chromatophilic erythroblasts (RII) stage in the BM and spleen (Figures S2A and S2B). Histological analysis showed normo- to hypercellular BM with more than 90% myeloid blast infiltration with significant nuclear atypia and atypical mitoses and apoptosis (Figure 2I), consistent with the diagnosis of AML.

### Outgrowth of d715-RHD AML without CSF3 Administration

To interrogate whether d715-RHD-derived AML was still dependent on CSF3 administration, we transplanted leukemic cells into tertiary recipients that were subsequently treated with PBS or CSF3 (n = 4 per group; Figure 2J). GFP^+^ cells in the PB were detectable from 10 weeks until the end of the experiment and increased over time in PBS- and CSF3-treated groups with similar kinetics (Figures 2K and 2L). c-Kit^+^ AML cells weakly expressed lineage markers (Figure 2L) but retained their myeloblast morphology (Figure 2M). The BM, spleen, and liver contained equivalent numbers of GFP^+^ cells independent of CSF3 treatment (Figure 2N), confirming that the outgrowth of AML no longer depended on CSF3 administration. Immunophenotyping revealed that, in addition to CD11b, the leukemic blasts co-expressed CD3, suggestive of a mixed myeloid/T-lymphoid phenotype (Figure 2O). As expected, all mice developed symptoms of AML, including severe anemia and a block of erythropoiesis in the BM and spleen (Figures S2C–S2E; data not shown). Although significant numbers of GFP^−^ CD71^intermediate/low^Ter119^+^ poly/ortho-chromatophilic erythroblasts and enucleated erythrocytes (RIII/RIV) were found in the liver, these cells did not compensate for the anemia (Figures S2C, S2F, and S2G).

### Leukemic Progression in d715-RHD Mice Is Accompanied by Elevated Pro-inflammatory Cytokine Responses

**Figure 3:**
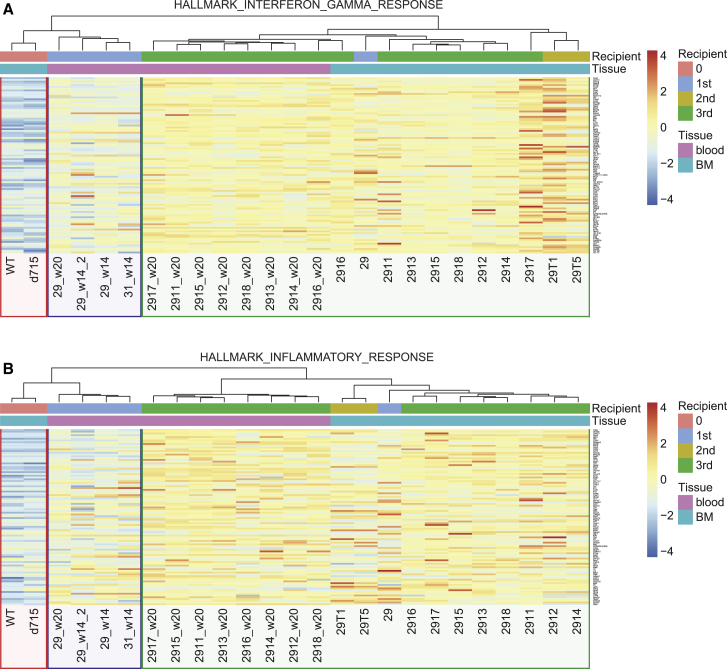
Elevated Inflammatory Transcriptomes Associated with Leukemic Progression in d715-RHD Mouse Models (A and B) Heatmap showing unsupervised clustering and expression of the top 100 differentially expressed transcripts in BM and PB samples of control, pre-leukemic, and leukemic LK/c-Kit^+^ cells (A) associated with IFN-γ signaling and (B) an inflammatory response.

To determine which signaling pathways changed during the sequential steps of transformation, we performed transcriptome profiling on FACS-purified BM- or PB-derived LK cells (1) prior to transplantation, (2) from d715-RHD CSF3-treated primary recipients (pre-leukemic), and (3) c-Kit^+^ cells from leukemic (secondary and tertiary recipient) mice (because these cells co-express lineage markers). Unsupervised clustering based on gene sets derived from GSEA-defined hallmark pathways revealed that LK cells from non-leukemic (t = 0, red), pre-leukemic (primary recipients, blue), and c-Kit^+^ cells from leukemic (secondary and tertiary recipients, green) mice clustered based on interferon γ (IFN-γ)- (Figure 3A), inflammatory- (Figure 3B), and tumor necrosis factor alpha (TNF-α)/nuclear factor κB (NF-κB)-responses (data not shown), which increased with progressive stages of transformation.

### Comparison of the Mouse Model with Clinical SCN-AML

**Figure 4:**
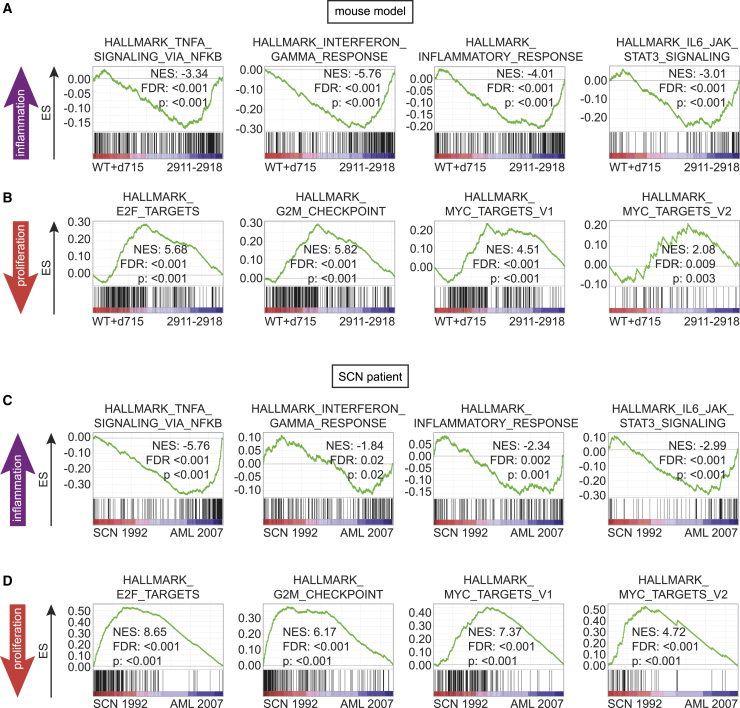
Leukemic Progression of the d715-RHD Mouse Model and SCN (SCN-AML) Is Associated with Increased Inflammatory and Decreased Proliferative Signaling (A and B) GSEA analyses comparing control (WT and d715 transcriptional data combined, n = 2) versus leukemic (tertiary recipients 2911–2918, n = 8) LK/c-Kit^+^ cells showing (A) upregulation of inflammatory signaling and (B) downregulation of proliferative pathways in leukemic mice. (C and D) GSEA comparing CD34^+^ cells from the SCN phase (1992) and the SCN-AML phase (2007), showing (C) increased inflammatory pathways and (D) downregulated proliferation signatures. ES, enrichment score; NES, normalized ES; FDR, false discovery rate. See also Figure S3 and Tables S1 and S2.

To determine whether the gene expression changes in the mouse model mimic the clinical progression of SCN to AML, we took advantage of bio-banked samples of a previously reported *ELANE* mutant-SCN patient who received life-long CSF3 therapy and acquired *CSF3R* (d715) and *RUNX1* (D171N) mutations identical to our mouse model.^10^ GSEA comparisons in the mouse showed that activation of inflammation-associated pathways TNF-α via NF-κB signaling, IFN-γ, inflammatory response, and interleukin-6 (IL-6)/JAK/STAT3 signaling were significantly elevated in leukemia samples relative to non-leukemic LK populations (the expression data of WT and *Csf3r*-d715 were combined and compared with the combined data of the eight tertiary transplant recipients; Figure 4A). On the other hand, canonical proliferative hallmark signatures (E2F, G2M checkpoint, and MYC), initially upregulated in the premalignant state conferred by the activated CSF3R-d715 and RUNX1-RHD (Figure 1F), were significantly blunted in the leukemic samples (Figure 4B).

Transcriptome analysis of FACS-purified CD34^+^ cells from the neutropenic phase (SCN 1992) and the AML phase (SCN-AML 2007) showed markedly similar hallmark signature patterns compared with the mouse model: upregulation of TNF-α via NF-κB signaling, IFN-γ and inflammatory responses, and IL6/JAK/STAT3 signaling pathways (Figure 4C) and downregulation of the proliferation signatures E2F, G2M checkpoint, and MYC (Figure 4D). GSEA comparing the CD34^+^ cells of the SCN-AML phase with 3 healthy controls showed the same transcriptional alterations (Figures S3A and S3B).

In addition, we found 55 of 241 significantly (p < 0.05) upregulated transcripts in SCN-AML samples overlapping with significantly (q < 0.05) upregulated transcripts in mouse AML samples (Table S1), whereas 53 of the 188 downregulated transcripts were overlapping (Table S2). These data furnish a rich substrate for further studies of the critical downstream mechanisms contributing to leukemic progression. For instance, *ANXA2*, *CDKN1A*, *CISH*, *DUSP1*, *FOS*, and *IL10RA* are prominent inflammatory genes that were commonly upregulated in mouse AML and patient SCN-AML samples (Table S1). Similarly, genes that are downregulated merit further study in the context of inflammation-driven leukemogenesis (Table S2).

### Whole-Exome Sequencing Identifies a Mutation in *Cxxc4* as a Somatic Clonal Driver for Progression to AML

**Figure 5:**
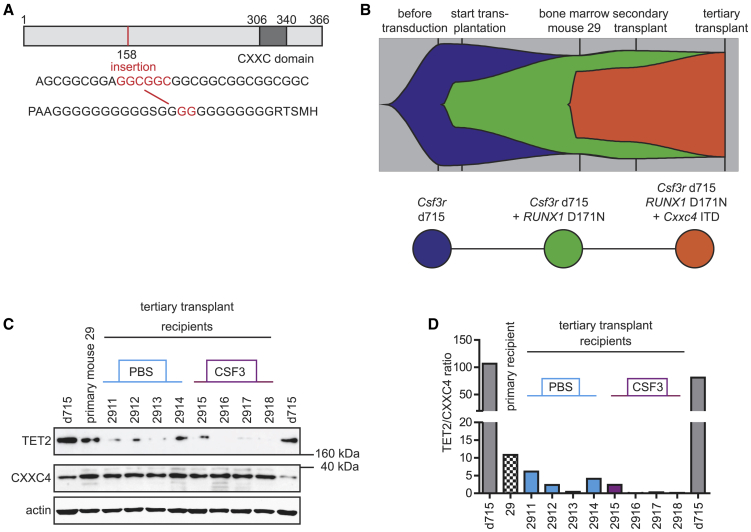
*Cxxc4* Mutations and Expression of CXXC4 and TET2 Proteins in AML Cells (A) Schematic overview of the CXXC4 gene, showing acquisition of a *Cxxc4-*ITD in mouse leukemic cells. (B) Fish plot illustrating the clonal pattern of leukemia development. (C) Immunoblot showing elevated CXXC4 protein levels (36.44 kDa) and reduced TET2 protein levels (212.13 kDa) in pre-leukemic and leukemic samples derived from primary recipient 29 (biological replicates). (D) Quantification of TET2/CXXC4 ratios. See also Figure S4.

Because we discriminated a pre-leukemic stage in our mouse model, we asked whether leukemic progression was caused by acquisition of additional driver mutations. To address this, we performed whole-exome sequencing on AML cells from one secondary recipients (29T1) and eight tertiary recipients (2911–2918). These leukemic mice harbored a relatively small number of newly acquired somatic mutations that were mostly subclonal and varied between individual mice (data not shown). The single mutation present in all leukemic mice was a clonal heterozygous abnormality (VAF, 0.52 ± 0.06) characterized by a 6-nt (GGCGGC) internal tandem duplication (ITD) in *Cxxc4*/*Idax*, introducing 2 glycine residues at amino acid position 158 of the WT protein (Figure 5A). This mutation already appeared in a subclone (VAF, 0.27) in primary recipient mouse 29 and expanded in the secondary and tertiary recipients, in which all AML cells harbored the *Csf3r*-d715, *RUNX1*-RHD, and *Cxxc4*-ITD mutations (Figure 5B). Notably, *Csf3r-*d715 donor BM samples did not contain any detectable reads with *Cxxc4*-ITD mutations, indicating that the mutation was acquired *de novo* as a somatic driver mutation during leukemic transformation (Figure 5B).

### The *Cxxc4*-ITD Mutation Correlates with Elevated CXXC4 and Reduced TET2 Protein Levels

To assess the effects of the *Cxxc4* mutation, we first determined its effect on protein expression. The predicted molecular weight of WT mouse CXXC4 is 36.4 kDa. Immunoblotting confirmed the presence of this CXXC4 isoform in *Csf3r*-d715 BM cells (Figure 5C) and showed 7-fold higher expression levels (7.25 ± 0.64) in pre-leukemic and leukemic samples harboring the *Cxxc4* mutation. *CXXC4*/*IDAX* is genetically closely linked to *TET2* and was originally encoded within the ancestral *TET2* gene before it became a separate gene during evolution.^11^ CXXC4 is an inhibitor of Wnt/β-catenin signaling.^12^ Suggestive of elevated CXXC4 activity, transcriptome analysis using GSEA showed reduced Wnt/β-catenin signaling in *Cxxc4*-ITD-expressing leukemia samples (2,911–2,918) relative to WT *Cxxc4* BM LK cells (data not shown). More striking in the context of leukemic progression, it was shown that CXXC4 activates proteolytic degradation of TET2 through a mechanism involving caspases 3 and 8.^11^ Indeed, we found that TET2 protein levels were severely reduced in leukemic relative to normal samples (0.20 ± 0.08), whereas reduced expression of *Tet2* was not observed on a transcriptional level (Figure 5C; data not shown). Quantification of TET2/CXXC4 ratios illustrates the strong inverse relationship between TET2 and CXXC4 (ITD) protein levels (Figure 5D).

### *CXXC4* Mutations in Human AML

**Figure 6:**
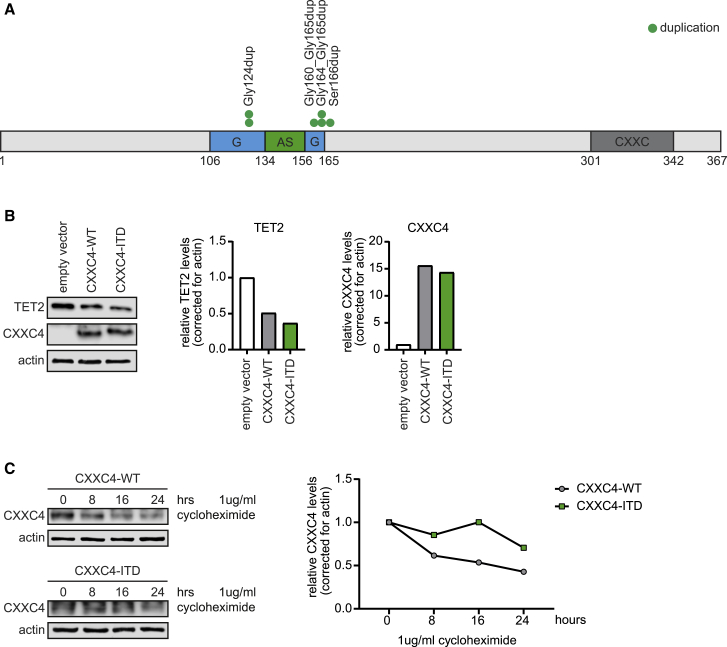
Human *CXXC4-*ITD Mutation Results in a More Stable CXXC4 Protein and Reduced TET2 Levels (A) Schematic overview of the human *CXXC4* gene, showing *CXXC4-*ITD mutations in *de novo* AML. (B) Overexpression of wild-type (WT) or the CXXC4-ITD (duplication of glycines 160–165) in K562 cells shows a subsequent reduction in TET2 protein levels by immunoblot. (C) Representative image and quantification of an immunoblot, showing increased stability of the CXXC4-ITD protein upon prolonged treatment with 1 μg/mL cycloheximide. See also Figure S4.

To determine whether *CXXC4*-ITD mutations similar to those identified in the mouse model are relevant for human disease, we analyzed whole-genome sequencing data from 591 AML patients generated at the Munich Leukemia Laboratory (MLL), complemented by targeted sequencing of 87 consecutive AML patients from the HOVON102 clinical trial. The CXXC4 protein shows high homology between mouse and human (Figure S4). In total, 6 *CXXC4-*ITD mutations were found (Figure 6A). Interestingly, the insertion of 2 glycine residues caused by the ITD in our mouse model was also found in 2 *de novo* AML patients. Besides the tandem glycine insertions, smaller insertions comprising 1 glycine residue or larger ITDs comprising 6 glycine residues were found. Cross-referencing with the gnomAD database (v.2.1.1), which contains sequences from 141,456 unrelated individuals (https://gnomad.broadinstitute.org/), showed that the *CXXC4*-ITD was absent or detected in 10- to 140-fold lower frequencies than in our cohort. This suggests that some *CXXC4-*ITD mutations may be somatic, whereas others may be present in the germline and predispose to leukemia. The fact that these insertions were all located in or around the glycine repeats suggests that this region is important for protein expression and/or stability.

### The CXXC4-ITD Reduces TET2 Levels and Has a Prolonged Half-Life Relative to WT CXXC4 Protein

To determine how a patient-specific *CXXC4*-ITD (Gly160_165dup) exerts its enhanced effect on reducing TET2 levels relative to WT CXXC4, we lentivirally introduced both forms in K562 and HEK293T cells. Ectopic overexpression of WT CXXC4 as well as the CXXC4-ITD resulted in a significant reduction in TET2 protein levels (Figure 6B), confirming the observations of Ko et al.^11^ Because we observed increased CXXC4 protein levels in murine *Cxxc4-*ITD leukemic cell samples (Figure 5C), we wondered whether elevated protein levels are a result of prolonged protein stability of the CXXC4-ITD protein. Indeed, translation inhibition by cycloheximide to block *de novo* protein synthesis showed that the CXXC4-ITD was maintained at higher levels than WT CXXC4 (Figure 6C), providing a plausible explanation for its elevated abundance in the mouse leukemia cells.

### *CSF3R*-d715 *RUNX1*-RHD Increases Proliferative Signaling in CD34^+^CD45^+^ Cells Derived from Control iPSCs while Inducing Inflammatory Pathways in SCN iPSCs

A limitation of the mouse transplantation model is that it lacks the disease-causing mutation of *ELANE*-SCN. However, *Elane* mutations equivalent to those found in SCN patients do not cause neutropenia in murine models,^13^ precluding the possibility to analyze the additional effect of *ELANE* mutations in murine models. To model leukemic progression in human SCN, we introduced the *CSF3R-*d715 mutation by CRISPR-Cas9-mediated genome editing and the *RUNX1-*D171N mutation by lentiviral transduction (containing IRES-GFP; Figure S1C) in iPSCs from an *ELANE* mutant-SCN patient (Figures S5A–S5C) and performed RNA sequencing on GFP^+^ CD34^+^CD45^+^ hematopoietic progenitor cells (HPCs) derived from two independent experiments. First we checked, in control iPSCs, whether GFP^+^ *CSF3R*-d715 mutant HPCs recapitulated the findings of *in vitro* mouse experiments (Figure 1F). When comparing RUNX1-RHD cells with ev control cells, the expression pattern corroborated the mouse experiments; e.g., increased proliferation-related signaling (compare Figure S6A with Figure 1F; the leukemic mice shown in Figure 3 acquired an additional mutation in *CXXC4*, altering the transcriptome). In addition, we observed reduced inflammatory signaling, establishing that *RUNX1* mutations per se do not cause inflammatory signaling but, instead, are involved in gaining a proliferative advantage (Figure S6A).

In contrast, in *RUNX1* mutant-expressing cells derived from an *ELANE* mutant patient, a slight increase in inflammatory responses (e.g., IFN-γ signaling) was observed compared with ev controls, which was associated with leukemic progression in the mouse model and SCN-AML patient (compare Figure S6B with Figures 1F and 4). In line with the data from control cells and the *in vitro* mouse data, the proliferation-related signature G2M checkpoint and E2F targets were enriched upon expression of the RUNX1-RHD mutant in an *ELANE* mutant, *CSF3R*-d715 background (Figure S6B). However, MYC targets were not induced, and even inhibited, in *ELANE* mutant cells (Figure S6B). These data suggest that the SCN background already primes for leukemic progression, where the combination of *ELANE, CSF3R*, and *RUNX1* mutations already shows signs of tipping the balance between inflammatory and proliferative signaling, as seen during the leukemic progression in the mouse model and SCN patient.

### Analysis of 5mC/5hmC Levels in Mouse Leukemia Samples

Several recent studies have linked TET2 to inflammatory responses and connected loss of TET2 to inflammation-driven clonal hematopoiesis, pre-leukemia, and MDS.^14^, ^15^, ^16^, ^17^, ^18^, ^19^ The effects of TET2 on repressing inflammation have been assigned to its methylcytosine dioxygenase activity but also to its ability to recruit histone deacetylase (HDAC)-mediated repressor activity to pro-inflammatory genes.^17^^,^^19^ To investigate whether the *Cxxc4-*ITD and subsequent reduction in TET2 levels altered the 5-hydroxymethylcytosine (5hmC) and 5-methylcytosine (5mC) levels of the DNA, we performed quantitative mass spectrometry experiments on LK cells derived from leukemic mice.^20^ Surprisingly, we did not observe major alterations in the proportion of 5mC and 5hmC nucleotides compared with LK cells from WT or d715 mice (Figure S7A), suggesting that the reduction of TET2 levels did not alter methylcytosine dioxygenase activity. This might be due to technical limitations, a redundancy with other methylcytosine dioxygenases,^21^ or the fact that reduced TET2 levels were not restrictive for its enzymatic activity. On the other hand, this could suggest that a noncanonical function of TET2, such as its ability to recruit HDAC activity to chromatin, rather than its enzymatic activity was mainly affected by the *Cxxc4-*ITD.

### *CSF3R*-d715 *RUNX1*-RHD Cells Switch Transcriptional Signatures from Proliferative to Inflammatory upon HDAC Inhibition in Human iPSC Models

**Figure 7:**
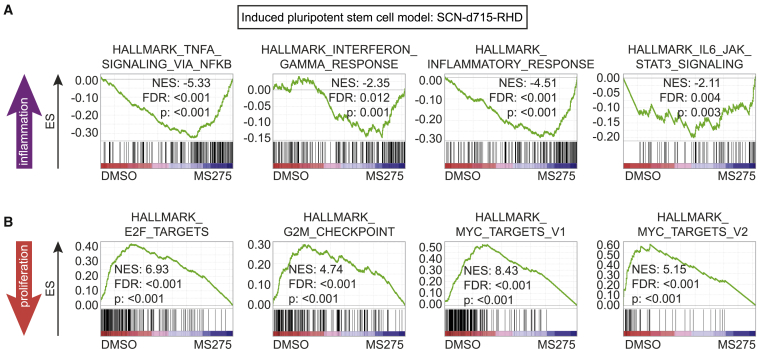
The HDAC Inhibitor MS275 Induces Inflammatory Signatures in SCN iPSC-Derived CD34^+^CD45^+^ Cells (A and B) GSEAs comparing CD34^+^CD45^+^*CSF3R-*d715 *RUNX1*-RHD cells from *ELANE* mutant SCN cells treated with DMSO or the HDAC inhibitor MS275, derived from two independent experiments (biological replicates), showing (A) induced inflammatory signaling and (B) reduced proliferative signatures in MS275-treated cells. See also Figure S5.

We next aimed to functionally validate the findings from leukemic mice in a human SCN-derived iPSC model expressing the truncated *CSF3R*-d715 and the *RUNX1-*RHD mutant. An obvious experiment would be to introduce the *CXXC4-*ITD in these cells by CRISPR-Cas9-mediated genome editing, but this was technically infeasible because of the high GC repeat content in the target region. Instead, to discriminate between the two functions of TET2 proposed above, we wanted to find out how inhibition of its enzymatic activity by administration of octyl-(R)-2hydroxyglutarate (2HG) and its ability to recruit HDAC activity by administration of the HDAC inhibitor MS275 (entinostat) affected the transcriptional profile of *CSF3R*-d715 *RUNX1*-RHD expressing SCN-iPSC-derived CD34^+^CD45^+^ HPCs. In line with the unaltered 5hmC levels in mouse AML cells, hallmark analyses showed that 2HG did not induce an inflammatory profile as observed in *Cxxc4* mutant mouse AML cells (Figure S7B). In contrast, HDAC inhibition with MS275 ignited inflammatory signaling while inhibiting the proliferative signatures in control and *ELANE* mutant cells, recapitulating the signaling profiles of *Cxxc4* mutant mouse AML cells and SCN-AML patient material (Figures 7A and 7B; data not shown). Although HDAC inhibition may also affect pathways independent of TET2, these data fit into a model of leukemic progression in which TET2 represses inflammatory signaling through its ability to recruit HDACs to pro-inflammatory genes,^17^^,^^19^ whereas DNA hydroxyl methylation had little or no effect. This model may apply specifically to the context of leukemic progression of SCN involving acquisition of *CSF3R* and *RUNX1* mutations but could also be relevant to other forms of AML (e.g., with *RUNX1* mutations and/or secondary to BM failure syndromes other than SCN).

## Discussion

In this study, we investigated how mutations in *CSF3R* and *RUNX1*, frequently associated with leukemic progression of SCN in conjunction with CSF3 treatment,^1^ contribute to AML development. By developing a mouse model with the most recurrent *CSF3R* and *RUNX1* mutations, we found that this combination resulted in accumulation of myeloblasts and myeloid colony-forming progenitors in PB, which persisted for 25+ weeks while on sustained CSF3 treatment. Although myeloblasts were found in PB, mice did not reveal signs of overt AML. These findings agree with clinical observations in an *ELANE* mutant SCN patient with *CSF3R* and *RUNX1* mutations (in the absence of other somatic defects) who showed CSF3-dependent expansion of a *CSF3R/RUNX1* mutant myeloblast clone that spontaneously disappeared after CSF3 treatment was stopped.^6^ These findings heralded that full malignant transformation of SCN to AML requires additional defects. This was confirmed in serial transplantation experiments in which we identified a clonal mutation in *Cxxc4* as a driver of AML development. This mutation resulted in increased levels of CXXC4 protein. CXXC4 has been recently shown to control caspase-mediated degradation of TET2,^11^ and, in agreement with this, TET2 levels were severely reduced in AML cells.

*CXXC4* mutations were also found in a minority of *de novo* AML patients. At what frequencies *CXXC4* mutations are present in SCN-AML or other forms of secondary AML and whether they coincide with acquisition of other mutations (e.g., in *RUNX1*) is currently under study. The possibility that mutations in other genes affecting the TET2 pathway will have similar consequences for development of SCN-AML must be considered in this context. In agreement with this, mutations in *SUZ12*, a component of EZH2-containing polycomb repressor complex 2 (PRC2), which inhibits CXXC4 expression,^22^ have been detected in the SCN-AML patient used for this study and others.^6^^,^^10^ Mutations in *ASXL1*, also recurrent in SCN-AML, may have similar effects and have recently been functionally connected to leukemic transformation in conjunction with *RUNX1* mutations.^23^^,^^24^ These findings are consistent with the concept that reduced TET2 levels contribute to leukemic progression of SCN.

Another key finding that emerged from our study concerns the connection between inflammation, clonal expansion, and leukemic progression of SCN. A role for inflammation in regulating hematopoietic stem cell fate in normal and several disease-related conditions, including MDS, has been suggested previously.^15^ Some studies showed that pro-inflammatory cytokines initially activate stem cells to exit from a dormant state but, when chronically active, cause loss of stem cell fitness, which eventually results in age-related BM failure.^25^, ^26^, ^27^ Importantly, TET2 has been shown to repress pro-inflammatory cytokine genes in macrophages and dendritic cells, in part by recruitment of HDAC2,^19^ whereas, inversely, loss of *Tet2* fosters an inflammatory state characterized by upregulation of IL-6 and TNF-α.^16^^,^^28^^,^^29^ More recent studies in *Tet2*-deficient mice showed that inflammation drives clonal expansion of pre-leukemic myeloid precursors.^14^^,^^17^^,^^30^ Taken together, these findings fit into a model where reduction of TET2 levels through gene disruption or downregulation of expression induces an inflamed state. Acquisition of a *RUNX1* mutation would then stimulate outgrowth of a malignant clone that evolves into AML.

Although the transcriptome analysis of mouse AML cells, the SCN-AML patient sample, and HDAC inhibitor-treated SCN iPSC-derived CD34^+^CD45^+^ cells showed elevated IL-6-, IFN-γ-, and TNF-α-mediated inflammatory responses, increased transcription of these cytokine genes themselves was not seen (data not shown). This could suggest that the inflammatory responses were activated by a cell-extrinsic rather than a cell-intrinsic (autocrine) stimulus. Because these studies were done on immature CD34^+^ or c-Kit^+^ fractions, it is possible that affected macrophages and/or dendritic cells derived from these cells and/or environmental cells, e.g., endothelial cells, produced elevated levels of pro-inflammatory cytokines, inducing an inflammatory state in immature progenitor populations in a paracrine manner. An alternative possibility is that the genomic damage underlying SCN itself activates inflammatory signaling, e.g., IFN-γ-induced pathways, which is aggravated when TET2 levels are reduced. For instance, increased IFN-γ signaling has been reported in the context of DNA double-strand breaks induced by etoposide treatment or X-ray irradiation of cells.^31^^,^^32^

In conclusion, this study provides insights into sequential steps in leukemic progression of SCN and identifies pro-inflammatory mechanisms as important mediators of this process. Questions that still need to be addressed are how mutant neutrophil elastase proteins shape the “fertile ground” for acquisition of *CSF3R* mutations and how CSF3 treatment contributes to reshaping this into the cellular state in which elevated inflammatory responses are induced and tolerated and *RUNX1* mutations can be acquired, resulting in progression to AML. Resolving these issues holds promise for clinical management of SCN, and possibly other leukemia predisposition syndromes, with the objective to avoid or interfere early in the malignant transformation of these conditions.

### Limitations of Study

Although *CSF3R/RUNX1/CXXC4* mutant cells could repopulate and form AML in multiple secondary (n = 6) and tertiary (n = 8) recipients, the *CXXC4-*ITD mutation originally arose in a single primary recipient (1/9), who acted as a donor for subsequent retransplantation studies. Hence, rather than being frequently involved in the leukemic progression of SCN, the *CXXC4-*ITD mutation should be taken as evidence that downregulation of TET2 and the associated upregulation of inflammatory signaling contribute to malignant transformation in conjunction with the *CSF3R* and *RUNX1* mutations. Supporting a role of *CXXC4* mutations in human disease, *CXXC4-*ITD mutations very similar to those detected in the mouse model were found in a subset of *de novo* AML patients, some but not all of which are found in the healthy population, but at much lower frequencies. Further studies are needed to address whether *CXXC4* mutations in human AML are somatic or germline. Studies to unravel co-occurrence with other mutations in, e.g., *RUNX1* or *TET2* could provide further insight into the pathogenic mechanism of *CXXC4* alterations. Inhibition of the HDAC-recruiting function of TET2 with the HDAC inhibitor MS275 provides a possible mechanism of how reduced TET2 levels can induce inflammatory signaling, but this inhibitor may also affect pathways independent of TET2. How TET2 regulates inflammation at specific gene targets and how *RUNX1* mutations affect this process (e.g., by enabling pre-leukemic cells to escape from inflammation-mediated exhaustion) merits further study.

## STAR★Methods

### Key Resources Table

**Table:** 

REAGENT or RESOURCE	SOURCE	IDENTIFIER
**Antibodies**		

Sca1-Pacific blue	Biolegend	Cat# 108120; RRID:AB_493273
CD34-PE	Biolegend	Cat# 128609; RRID:AB_2074602
CD48-PE	Biolegend	Cat# 103405; RRID:AB_313020
CD150-PE.Cy7	Biolegend	Cat# 115913; RRID:AB_439796
TER119-APC	Biolegend	Cat# 116211; RRID:AB_313712
CD117-APC	BD Biosciences	Cat# 553356; RRID:AB_398536
CD16/32-APC.Cy7	BD Biosciences	Cat# 560541; RRID:AB_1645229
CD11b-PE	BD Biosciences	Cat# 553311; RRID:AB_394775
Gr1-APC	BD Biosciences	Cat# 553129; RRID:AB_398532
CD3-PE.Cy7	BD Biosciences	Cat# 560591; RRID:AB_1727462
CD71 PE	BD Biosciences	Cat# 553267; RRID:AB_394744
CD19-AF700	Life	Cat# 56-0193-82; RRID:AB_837083
CD117-PerCpCy5.5	SONY	Cat# 1129115
murine lineage detection kit	BD Biosciences	Cat# 559971; RRID:AB_10053179
Streptavidin-pacific orange	Fisher Scientific	Cat# S32365
SSEA4-PE	Biolegend	Cat# 330406; RRID:AB_1089206
Tra-1-60-AF647	BD Biosciences	Cat# 560122; RRID:AB_1645448
CD34-PE	BD Biosciences	Cat# 345802; RRID:AB_400078
CD45-BV421	BD Biosciences	Cat# 563879; RRID:AB_2744402
7AAD	Life	Cat# A1310
DAPI	Fisher Scientific	Cat# D1306; RRID:AB_2629482
Anti-human AML1	Cell Signaling Technology	Cat# 4334; RRID:AB_2184099
Anti-mouse/human AML1	Cell Signaling Technology	Cat# 8529; RRID:AB_10950225
CXXC4	Novus Bio	Cat# NBP1-76491; RRID:AB_11028768
TET2	Abcam	Cat# ab94580; RRID:AB_10887588

**Bacterial and Virus Strains**		

pMY-RUNX1 retroviral vectors	Goyama et al.^35^	N/A
Lentiviral CSI vector	Brian Duncan	N/A
pBabe vector	Morgenstern and Land^36^	RRID:Addgene_1764

**Biological Samples**		

Bone marrow and blood samples from SCN and SCN-AML patient	Beekman et al.^10^	N/A

**Chemicals, Peptides, and Recombinant Proteins**		

CSF3 (Filgrastim)	Zarzio	N/A
Ciprofloxacin	Centrafarm	Cat# 8714632211261
Geltrex LDEV-Free Reduced Growth Factor Basement Membrane Matrix	Thermo Fisher Scientific	Cat# A1413302
mTeSR1	STEMCELL Technologies	Cat# 85850
TransIt transfection reagent	Mirus	Cat# MIR 2300
IL-6	Tebu-bio	Cat# 167200-06-B
SCF	Reprotech	Cat# 250-03
GM-CSF	Tebu-bio	Cat# 167315-03-B
Retronectin	TaKaRa	Cat# T100B
Methylcellulose	STEMCELL Technologies	Cat# M3234
CellGro GMP serum-free stem cell growth medium	CellGenix	Cat# 20802-0500
TPO	Reprotech	Cat# 315-1
IGF	R&D systems	Cat# 792-MG
FGF	Reprotech	Cat# 450-33A
Puromycin	Sigma Aldrich	Cat# P7255
FuGENE HD	Promega	Cat# E2311
Hexadimethrine bromide (polybrene)	Sigma	Cat# 107689
Cycloheximide	Merck	Cat# C4859
DMSO	Sigma	Cat# D2650
Octyl-(R)-2HG	Sigma-Aldrich	Cat# SML2200
MS275	Santa Cruz	Cat# sc-279455A
TRIzol	Thermo Fisher Scientific	Cat# 15596018
GenElute™−LPA	Sigma-Aldrich	Cat# 56575

**Critical Commercial Assays**		

Biotin Mouse Hematopoietic Progenitor (Stem) Enrichment Set	Thermo Fisher Scientific	Cat# 558451
STEMdiff Hematopoietic Kit	STEMCELL Technologies	Cat# 053108
SMARTer Ultra Low Input RNA kit for sequencing	Clontech	Version 3 Cat# 634851
		Version 4 Cat# 634891
TruSeq Nano DNA Sample Preparation kits	Illumina	Cat# 20015964
SeqCap EZ HyperPlusCap workflow	Roche	Cat# 6740278001
MiSeq V2 Nano kit	Illumina	Version 2 Cat# MS-103-1001

**Deposited Data**		

FastQ files *in vitro* mouse RNA seq	This paper	ArrayExpress: E-MTAB-9373
FastQ files *in vivo* mouse RNA seq	This paper	ArrayExpress: E-MTAB-9377
FastQ files mouse DNA seq	This paper	ArrayExpress: E-MTAB-9376
Count files human RNA seq	This paper	ArrayExpress: E-MTAB-9381
Count files iPSC RNA seq	This paper	ArrayExpress: E-MTAB-9375
Count files iPSC inhibitor RNA seq	This paper	ArrayExpress: E-MTAB-9374

**Experimental Models: Cell Lines**		

Human: *ELANE-*mutant SCN iPSC	This paper	N/A
Human: control iPSC	This paper	N/A
K562	ATCC	Cat# CCL-243
HEK293T	ATCC	Cat# CRL-1573

**Experimental Models: Organisms/Strains**		

Mouse: FVB/n Csf3r-d715	Hermans et al.^33^	N/A
Mouse: FVB/n wt: FVB/NHanHsd	Envigo	Cat# 862-NL

**Oligonucleotides**		

CXXC4 forward primer: AGGGGATAAGGTGGAGAGGA	This paper	N/A
CXXC4 reverse primer: CCCCTGGAACTGCGACAA	This paper	N/A

**Recombinant DNA**		

pCL-eco	Naviaux et al.^37^	RRID:Addgene_12371
pSPAX2	Didier Trono	RRID:Addgene_12260
MD2.G	Didier Trono	RRID:Addgene_12259
px330	Cong et al.^40^	RRID:Addgene_42230

**Software and Algorithms**		

GraphPadPrism 5.0c	GraphPad Software Inc.	RRID:SCR_002798
Adobe Illustrator CC 2018	Adobe Systems Inc.	RRID:SCR_010279
FlowJo V10	TreeStar Inc.	RRID:SCR_008520
CASAVA	Illumina	RRID:SCR_001802
FastQC	Babraham bioinformatics	RRID:SCR_014583
MultiQC		RRID:SCR_014982
STAR aligner	Dobin et al.^41^	RRID:SCR_015899
Cufflinks	Trapnell et al.^42^	RRID:SCR_014597
HTSeq-count	Anders et al.^43^	RRID:SCR_011867
DESeq2	Love et al.^44^	RRID:SCR_015687
R.		RRID:SCR_001905
GSEA	Mootha et al.^45^; Subramanian et al.^46^	RRID:SCR_003199

### Resource Availability

#### Lead Contact

Further information and requests for resources and reagents should be directed to and will be fulfilled by the Lead Contact, Ivo P. Touw (i.touw@erasmusmc.nl).

#### Materials Availability

Viral constructs and iPSC lines generated in this study will be made available on request, but we may require a payment and/or a completed Materials Transfer Agreement if there is potential for commercial application.

#### Data and Code Availability

The RNA sequencing dataset comparing the effect of the introduction of RUNX1-D171N (RHD) in *Csf3r*-d715 mice bone marrow cells generated during this study, as shown in Figure 1, is available at ArrayExpress: E-MTAB-9373.

The RNA sequencing dataset comparing different stages of leukemic progression in a mouse model of SCN generated during this study, as shown in Figures 3 and 4 and Tables S1 and S2, is available at ArrayExpress: E-MTAB-9377.

The RNA sequencing dataset comparing the transcriptome of a SCN patient who progressed to AML (SCN-AML) with the SCN phase and healthy controls generated during this study, as shown in Figures 4 and S3 and Tables S1 and S2, is available at ArrayExpress: E-MTAB-9381.

The DNA sequencing dataset comparing different stages of leukemic progression generated during this study, as shown in Figure 5, is available at ArrayExpress: E-MTAB-9376.

The RNA sequencing dataset comparing the effect of the introduction of RUNX1-D171N (RHD) in control- or SCN -*CSF3R*-d715 hematopoietic progenitor cells (HPCs) generated during this study, as shown in Figure S6, is available at ArrayExpress: E-MTAB-9375.

The RNA sequencing dataset comparing SCN *CSF3R*-d715 RUNX1-D171N (RHD) HPCs treated with the HDAC inhibitor MS275, octyl-(R)-2hydroxyglutarate (2HG) or solvent control (DMSO) generated during this study, as shown in Figures 7 and S7, is available at ArrayExpress: E-MTAB-9374.

### Experimental Model and Subject Details

#### Mice

All animals were kept according to Erasmus MC guidelines and experiments were performed under CCD license EMCAVD101002017869. FVB/n Csf3r-d715 knock-in mice,^33^ were bred at the Erasmus MC animal facility under specific pathogen free conditions and the mice were genotyped by PCR. FVB/n wild-type (wt) control mice were purchased from Envigo, Horst, the Netherlands. All animals used were male, single housed, 10-12 weeks old at the start of the experiment, and randomly assigned to experimental groups.

Lentivirally transduced *CSF3R*-d715 lineage negative bone marrow cells were transplanted 72 hours after the first transduction. GFP expression was determined by flow cytometry and 1x10^5^ GFP positive cells were transplanted, together with their GFP negative counterparts and 1x10^5^ spleen cells, into 8.5 Gy irradiated (Cesium-137, GammaCell GC40) FVB/n wt recipients. Secondary transplant FVB/n wt recipients received 5 Gy irradiation and 3x10^6^ total bone marrow cells together with 1x10^5^ spleen cells. The tertiary FVB/n wt recipients were irradiated with 8.5 Gy (IBL637 Cs-137) and received 2x10^5^ total bone marrow cells together with 1x10^5^ spleen cells.

Mice were injected subcutaneously three times a week with 100 μg/kg CSF3 (Filgrastrim, Zarzio) or vehicle control (PBS). Treatment was started five weeks after transplantation in the primary recipients. The secondary recipients started CSF3 treatment four weeks after transplantation, while the tertiary transplant recipients received CSF3 or PBS treatment two weeks after transplantation. The tertiary recipients received a higher dose of CSF3 (200 μg/kg). From the start of the experiment, all mice received 50 μg/ml of Ciprofloxacin in the drinking water.

#### Patient samples

Ficoll-gradient separated bone marrow and blood cells were obtained and frozen according to established procedures for viable cell cryopreservation. The study was performed under the permission of the Institutional Review Boards of the Erasmus MC, the Netherlands (registration number MEC-2008-387 for biobanking and MEC-2012-030 for the genetic analysis of leukemic progression in SCN patients).

#### Generation of iPSC

Bone marrow fibroblasts cultured from a healthy control (22 year old female) and a SCN patient (3 months old female) harboring *ELANE* mutation (chr19:852986A > T) I60F were reprogrammed at the iPSC core facility of the Erasmus MC using a previously described protocol.^34^ Cells were cultured in mTeSR1 (STEMCELL Technologies) on Geltrex LDEV-Free Reduced Growth Factor Basement Membrane Matrix (Thermo Fisher Scientific) at 37°C and 5% CO_2_, and were regularly checked for pluripotency and correct karyotype (Figure S5A).

### Method Details

#### Bone marrow isolation and lineage depletion

Bone marrow (BM) was isolated from femurs, tibias and sternum of the mice. Cells were harvested by crushing bones in a mortar, and the harvested marrow depleted from erythrocytes by Stem-Kit lysing solution (Beckman Coulter). The early stem and progenitor compartment was enriched using the Biotin Mouse Hematopoietic Progenitor (Stem) Enrichment Set (Thermo Fisher Scientific), according to the manufacturer’s protocol.

#### Viral expression constructs, virus production and transduction of BM cells

pMY-RUNX1 retroviral vectors^35^ were obtained from Gang Huang (Cincinnati Children’s Hospital, Cincinnati). RUNX1b mutant 4518 (D171N, RHD mutant) insert was subsequently cloned into the pBabe vector^36^ and used in *in vitro* mouse studies. For the *in vivo* and iPSC studies, the RUNX1 mutant was inserted into the lentiviral CSI vector (kind gift from Brian Duncan, University College London). The lentiviral CSI vector was also used to express CXXC4-wild-type and CXXC4-ITD (Glycine 160-165 duplication). All constructs were tested for correct introduction of inserts by enzymatic digestion and Sanger sequencing. Retroviral supernatants were harvested from HEK293T cells 48 hours after transfection with 5 μg of pBabe RUNX1-RHD mutant or empty vector (ev), together with 5 μg pCL-eco^37^ and 30 μL TransIt transfection reagent (Mirus). Lentiviral supernatants were obtained from HEK293T cells 48 hours after transfection with 4 μg of the CSI vector, 3 μg pSPAX2, 1 μg pMD2.G and 25 μL FuGENE HD (Promega). Viral supernatant and 1x10^6^ lineage depleted BM cells were incubated in RPMI medium (GIBCO) supplemented with 10 ng/ml each of murine interleukin-3 (IL-3), interleukin-6 (IL-6, Tebu-bio), stem cell factor (SCF, Preprotech) and granulocyte macrophage colony-stimulating factor (GM-CSF, Tebu-bio) on retronectin coated dishes (TaKaRa). Fresh virus supernatant was again added after 24 hours and transduced BM cells harvested another 48 hours later (72 hours after the first transduction).

#### SDS-PAGE/Immunoblotting

RUNX1 expression was determined using a RUNX1 antibody directed to human AML1 (4334, Cell Signaling Technology) or an antibody that recognized both human and mouse AML1 (8529, Cell Signaling Technology) in whole cell lysates. CXXC4 (NBP1-76491, Novus Bio) and TET2 (ab94580, Abcam) antibodies were used to determine both mouse and human protein levels in nuclear lysates. Preparation of lysates, and western blot analyses were performed as previously described.^38^

#### Murine colony assays

Lineage negative bone marrow cells from FVB/n mice harboring either a wt or a truncated CSF3R were isolated, and retrovirally transduced with either RUNX1 RHD mutant or an ev control. Cells were then seeded for colony formation (4x10^4^/ml) in methylcellulose (M3234, STEMCELL Technologies) with 50 ng/ml CSF3. Colonies were counted after 7 to 9 days of culture and replated if applicable.

#### Suspension cultures

1x10^5^ lineage depleted bone marrow cells per ml were plated after transduction into LODISH stem cell expansion medium. LODISH medium consisted of CellGro GMP serum-free stem cell growth medium (SCGM, CellGenix, Freiburg, Germany) supplemented with 10 ng/ml each of SCF, thrombopoietin (TPO, Preprotech), insuline-like growth factor (IGF, R&D systems), fibroblast growth factor (FGF, Preprotech) and with or without 50 ng/ml CSF3. Angiopoietin was harvested as conditioned medium of transfected HEK293T cells, and added to the culture medium in a 1:5 dilution.

#### Flow cytometry and cell sorting

Cellular composition of the liquid cultures and *in vivo* tissues were analyzed using flow cytometry. The following antibodies and recombinant proteins were used: Sca1-Pacific blue (PB), CD34-Phycoerythrin (PE), CD48-PE, CD150-PE.Cy7, Ter119 Allophycocyanin (APC)(all Biolegend), CD117-APC, CD16/32-APC.Cy7, CD11b-PE, Gr1-APC, CD3-PE.Cy7, CD71 PE (all BD Biosciences), CD19 Alexa Fluor 700 (AF700, Life) and CD117 PerCpCy5.5 (SONY). Lineage positive cells were detected using the murine lineage detection kit (BD Biosciences) subsequently stained with streptavidin-pacific orange (PO, Fisher Scientific).

SSEA4-PE (Biolegend) and Tra-1-60 Alexa Fluor 647 (AF647, BD Biosciences) were used for regular pluripotency screenings of the iPSCs. Hematopoietic induction of iPSC was assessed using CD34-PE and CD45-BV421 antibodies (both BD Biosciences). Dead cells were excluded by using 7-aminoactinomycin-D (7AAD, Life) or 4’,6-Diamidino-2-Phenylindole (DAPI, Fisher Scientific). Flow cytometry was performed using a BD LSRII flow cytometer (BD Biosciences). For subsequent cell sorting a FACSAria (BD Biosciences) was used. Analysis of FACS data was performed using FlowJo (TreeStar).

#### Blood, bone marrow, spleen and liver isolation from mice

Peripheral blood (PB) was obtained by cheek puncture. Upon sacrifice, BM was isolated from femurs and tibias of the mice. Cells were harvested by flushing the bones and subsequently filtered. Cells from spleen and liver were obtained by mashing parts of the organs through a filter.

#### Histological and morphological analyses

Mouse organs were fixed in 4% formaldehyde overnight, dehydrated and prepared for paraffin embedding, after which they were stained with Haemotoxylin and Eosin (H&E).

Cells were attached to glass slides using a Cytospin 4 (Thermo Scientific) according to the manufacturer’s protocol and stained with May-Grünwald Giemsa staining. Stainings were performed according to routine protocols.

#### Introduction of CXXC4 in K562/HEK293T and protein stability assays

Lentiviral supernatant containing either an ev control, CXXC4-wt or the CXXC4-ITD (duplication of Glycines 160-165) was added to K562 and HEK293T cells. Transduced cells were sorted for GFP^+^ expression and subsequently used. To test protein stability of CXXC4-wt or -ITD, we added 1 μg/ml of the translation inhibitor cycloheximide (Merck) and harvested the cells 0, 8, 16 or 24 hours after the start of the treatment.

#### Targeted sequencing of CXXC4

Custom CXXC4 amplicon sequencing was performed on patients of the HOVON102 trial using the Illumina PCR-based Custom Amplicon Library Preparation workflow. Forward primer AGGGGATAAGGTGGAGAGGA and reverse primer CCCCTGGAACTGCGACAA were used to amplify the Glycine repeat region of CXXC4. Libraries were paired-end sequenced with the MiSeq V2 Nano kit (Illumina) on the Illumina MiSeq. Bioinformatic analysis was performed as previously described.^39^

#### CRISPR/Cas9-mediated genome editing

To introduce the *CSF3R*-d715 mutation in iPSCs, 2x10^6^ cells were electroporated with 500 ng px330^40^ (Cas9 plasmid, Addgene plasmid #42230) and 1500 ng recombination template containing the d715 mutation and a puromycin selection cassette (Figure S5B), using the 4D-Nucleofector System (Lonza), program CA-137. Puromycin selection (300 ng/ml) started 48 hr after electroporation. Single clones were picked and screened for correct and heterozygous integration of the CSF3R mutation and pluripotency (Figures S5A and S5B).

#### Hematopoietic induction and colony assays from iPSC

Hematopoietic progenitor cells (HPCs, CD34^+^CD45^+^) were produced with the STEMdiff Hematopoietic Kit (STEMCELL Technologies) according to the manufacturer’s protocol. Floating cells were harvested at Day 12 of the protocol.

#### Lentiviral production and transduction of iPSC

Lentiviral supernatant was harvested at 24 and 48 hours after transfection of HEK293T cells with 4 μg of the CSI vector, 3 μg pSPAX2, 1 μg pMD2.G, and 25 μL FuGENE HD (Promega). Viral particles were 100x concentrated by ultracentrifugation (Optima XPN-80, Beckman Coulter) for 2 hours at 20.000 g and 4°C. Virus (25 μl/ml) and hexadimethrine bromide (polybrene, 4 μg/ml, Sigma) were added to the hematopoietic induction cultures 60 hours before harvesting the floating cells.

#### TET inhibitor experiments

DMSO (solvent control), 0.1 mM Octyl-(R)-2HG (Sigma-Aldrich) or 2 μM MS275 (Santa Cruz) was added for 16 hours to the hematopoietic induction cultures before harvesting the floating cells.

#### RNA isolation and RNA sequencing

RNA was isolated from FACS purified mouse LK or c-Kit^+^ cells, human CD34^+^ cells from the bone marrow (SCN phase, 1992) or blood (AML phase, 2007) or CD34^+^CD45^+^ HSCs derived from the iPSCs using TRIzol (Thermo Fisher Scientific) according to the manufacturer’s protocol, with the addition of GenElute™−LPA(Sigma-Aldrich). For the *in vitro* mouse experiments cDNA was generated with the SMARTer Ultra Low Input RNA kit for sequencing (version 3, Clontech), while version 4 of the SMARTer Ultra Low Input RNA kit for sequencing (Clontech) was used for the other samples. Sequencing libraries were generated using TruSeq Nano DNA Sample Preparation kits (Illumina), according to the low sample protocol and run on HiSeq 2500 or Novaseq 6000 instruments (Illumina).

#### Whole exome sequencing

100 ng genomic DNA was fragmented using enzymatic fragmentation and sample libraries were constructed following the SeqCap EZ HyperPlusCap workflow User’s Guide version 1.0 (Roche). Unique, dual index adapters (Integrated DNA technologies) were used for ligation. After ligation of adapters and an amplification step, exome target sequences were captured using in-solution oligonucleotide baits (SeqCap EZ Developer Library mm9_exome_L2R_D02). Amplified captured sample libraries were paired-end sequenced (2x100 cycles) on the HiSeq 2500 platform (Illumina).

#### Bioinformatics and statistics

Demultiplexing was performed using the CASAVA software (Illumina) allowing for one mismatch in the barcodes. Subsequently quality metrics were estimated (FastQC, Babraham bioinformatics & MultiQC; https://multiqc.info/) for all of the resulting fastq files. Afterward reads were aligned against the Mouse Transcriptome (Gencode m12)/Genome (mm10) or Human Transcriptome (Gencode v19)/Genome (hg19) using the STAR aligner.^41^ Abundance estimation was performed using Cufflinks^42^ (refSeq), and raw counts were measured with the HTSeq-count software set in union mode.^43^ Next the measured raw counts were used to create clustering and principle component plots and perform differential expression analysis both using DESeq2^44^ and R (https://www.r-project.org/). Finally gene set enrichment analysis, with the hallmark pathways H, was done using the GSEA software (https://www.gsea-msigdb.org/gsea/msigdb/genesets.jsp?collection=H).^45^^,^^46^

### Quantification and Statistical Analysis

Data are presented as mean ± SEM. Comparison of two groups was performed using unpaired t test. Statistical analyses were performed using GraphPad Prism 5.0c (GraphPad Software Inc., San Diego, CA) or DESeq2. A p value < 0.05 was considered significant.

## Supplemental Information

Document S1. Figures S1–S7 and Tables S1 and S2

Document S2. Article plus Supplemental Information

## References

[1] Skokowa J., Dale D.C., Touw I.P., Zeidler C., Welte K. (2017). Severe congenital neutropenias. Nat. Rev. Dis. Primers.

[2] Dale D.C., Bonilla M.A., Davis M.W., Nakanishi A.M., Hammond W.P., Kurtzberg J., Wang W., Jakubowski A., Winton E., Lalezari P. (1993). A randomized controlled phase III trial of recombinant human granulocyte colony-stimulating factor (filgrastim) for treatment of severe chronic neutropenia. Blood.

[3] Rosenberg P.S., Alter B.P., Bolyard A.A., Bonilla M.A., Boxer L.A., Cham B., Fier C., Freedman M., Kannourakis G., Kinsey S., Severe Chronic Neutropenia International Registry (2006). The incidence of leukemia and mortality from sepsis in patients with severe congenital neutropenia receiving long-term G-CSF therapy. Blood.

[4] Rosenberg P.S., Zeidler C., Bolyard A.A., Alter B.P., Bonilla M.A., Boxer L.A., Dror Y., Kinsey S., Link D.C., Newburger P.E. (2010). Stable long-term risk of leukaemia in patients with severe congenital neutropenia maintained on G-CSF therapy. Br. J. Haematol..

[5] Touw I.P. (2015). Game of clones: the genomic evolution of severe congenital neutropenia. Hematology (Am. Soc. Hematol. Educ. Program).

[6] Skokowa J., Steinemann D., Katsman-Kuipers J.E., Zeidler C., Klimenkova O., Klimiankou M., Unalan M., Kandabarau S., Makaryan V., Beekman R. (2014). Cooperativity of RUNX1 and CSF3R mutations in severe congenital neutropenia: a unique pathway in myeloid leukemogenesis. Blood.

[7] Sroczynska P., Lancrin C., Kouskoff V., Lacaud G. (2009). The differential activities of Runx1 promoters define milestones during embryonic hematopoiesis. Blood.

[8] Bee T., Liddiard K., Swiers G., Bickley S.R., Vink C.S., Jarratt A., Hughes J.R., Medvinsky A., de Bruijn M.F. (2009). Alternative Runx1 promoter usage in mouse developmental hematopoiesis. Blood Cells Mol. Dis..

[9] Miyoshi H., Ohira M., Shimizu K., Mitani K., Hirai H., Imai T., Yokoyama K., Soeda E., Ohki M. (1995). Alternative splicing and genomic structure of the AML1 gene involved in acute myeloid leukemia. Nucleic Acids Res..

[10] Beekman R., Valkhof M.G., Sanders M.A., van Strien P.M., Haanstra J.R., Broeders L., Geertsma-Kleinekoort W.M., Veerman A.J., Valk P.J., Verhaak R.G. (2012). Sequential gain of mutations in severe congenital neutropenia progressing to acute myeloid leukemia. Blood.

[11] Ko M., An J., Bandukwala H.S., Chavez L., Aijö T., Pastor W.A., Segal M.F., Li H., Koh K.P., Lähdesmäki H. (2013). Modulation of TET2 expression and 5-methylcytosine oxidation by the CXXC domain protein IDAX. Nature.

[12] Hino S., Kishida S., Michiue T., Fukui A., Sakamoto I., Takada S., Asashima M., Kikuchi A. (2001). Inhibition of the Wnt signaling pathway by Idax, a novel Dvl-binding protein. Mol. Cell. Biol..

[13] Grenda D.S., Johnson S.E., Mayer J.R., McLemore M.L., Benson K.F., Horwitz M., Link D.C. (2002). Mice expressing a neutrophil elastase mutation derived from patients with severe congenital neutropenia have normal granulopoiesis. Blood.

[14] Abegunde S.O., Buckstein R., Wells R.A., Rauh M.J. (2018). An inflammatory environment containing TNFα favors Tet2-mutant clonal hematopoiesis. Exp. Hematol..

[15] Barreyro L., Chlon T.M., Starczynowski D.T. (2018). Chronic immune response dysregulation in MDS pathogenesis. Blood.

[16] Cull A.H., Snetsinger B., Buckstein R., Wells R.A., Rauh M.J. (2017). Tet2 restrains inflammatory gene expression in macrophages. Exp. Hematol..

[17] Meisel M., Hinterleitner R., Pacis A., Chen L., Earley Z.M., Mayassi T., Pierre J.F., Ernest J.D., Galipeau H.J., Thuille N. (2018). Microbial signals drive pre-leukaemic myeloproliferation in a Tet2-deficient host. Nature.

[18] Shen Q., Zhang Q., Shi Y., Shi Q., Jiang Y., Gu Y., Li Z., Li X., Zhao K., Wang C. (2018). Tet2 promotes pathogen infection-induced myelopoiesis through mRNA oxidation. Nature.

[19] Zhang Q., Zhao K., Shen Q., Han Y., Gu Y., Li X., Zhao D., Liu Y., Wang C., Zhang X. (2015). Tet2 is required to resolve inflammation by recruiting Hdac2 to specifically repress IL-6. Nature.

[20] Kroeze L.I., Aslanyan M.G., van Rooij A., Koorenhof-Scheele T.N., Massop M., Carell T., Boezeman J.B., Marie J.P., Halkes C.J., de Witte T., EORTC Leukemia Group and GIMEMA (2014). Characterization of acute myeloid leukemia based on levels of global hydroxymethylation. Blood.

[21] An J., Rao A., Ko M. (2017). TET family dioxygenases and DNA demethylation in stem cells and cancers. Exp. Mol. Med..

[22] Lu H., Sun J., Wang F., Feng L., Ma Y., Shen Q., Jiang Z., Sun X., Wang X., Jin H. (2013). Enhancer of zeste homolog 2 activates wnt signaling through downregulating CXXC finger protein 4. Cell Death Dis..

[23] Gelsi-Boyer V., Brecqueville M., Devillier R., Murati A., Mozziconacci M.J., Birnbaum D. (2012). Mutations in ASXL1 are associated with poor prognosis across the spectrum of malignant myeloid diseases. J. Hematol. Oncol..

[24] Nagase R., Inoue D., Pastore A., Fujino T., Hou H.A., Yamasaki N., Goyama S., Saika M., Kanai A., Sera Y. (2018). Expression of mutant Asxl1 perturbs hematopoiesis and promotes susceptibility to leukemic transformation. J. Exp. Med..

[25] Essers M.A., Offner S., Blanco-Bose W.E., Waibler Z., Kalinke U., Duchosal M.A., Trumpp A. (2009). IFNalpha activates dormant haematopoietic stem cells in vivo. Nature.

[26] Pietras E.M. (2017). Inflammation: a key regulator of hematopoietic stem cell fate in health and disease. Blood.

[27] Walter D., Lier A., Geiselhart A., Thalheimer F.B., Huntscha S., Sobotta M.C., Moehrle B., Brocks D., Bayindir I., Kaschutnig P. (2015). Exit from dormancy provokes DNA-damage-induced attrition in haematopoietic stem cells. Nature.

[28] Hemmati S., Haque T., Gritsman K. (2017). Inflammatory Signaling Pathways in Preleukemic and Leukemic Stem Cells. Front. Oncol..

[29] Leoni C., Montagner S., Rinaldi A., Bertoni F., Polletti S., Balestrieri C., Monticelli S. (2017). *Dnmt3a* restrains mast cell inflammatory responses. Proc. Natl. Acad. Sci. USA.

[30] Cai Z., Kotzin J.J., Ramdas B., Chen S., Nelanuthala S., Palam L.R., Pandey R., Mali R.S., Liu Y., Kelley M.R. (2018). Inhibition of Inflammatory Signaling in Tet2 Mutant Preleukemic Cells Mitigates Stress-Induced Abnormalities and Clonal Hematopoiesis. Cell Stem Cell.

[31] Erdal E., Haider S., Rehwinkel J., Harris A.L., McHugh P.J. (2017). A prosurvival DNA damage-induced cytoplasmic interferon response is mediated by end resection factors and is limited by Trex1. Genes Dev..

[32] Brzostek-Racine S., Gordon C., Van Scoy S., Reich N.C. (2011). The DNA damage response induces IFN. J. Immunol..

[33] Hermans M.H., Ward A.C., Antonissen C., Karis A., Löwenberg B., Touw I.P. (1998). Perturbed granulopoiesis in mice with a targeted mutation in the granulocyte colony-stimulating factor receptor gene associated with severe chronic neutropenia. Blood.

[34] Warlich E., Kuehle J., Cantz T., Brugman M.H., Maetzig T., Galla M., Filipczyk A.A., Halle S., Klump H., Schöler H.R. (2011). Lentiviral vector design and imaging approaches to visualize the early stages of cellular reprogramming. Mol. Ther..

[35] Goyama S., Schibler J., Cunningham L., Zhang Y., Rao Y., Nishimoto N., Nakagawa M., Olsson A., Wunderlich M., Link K.A. (2013). Transcription factor RUNX1 promotes survival of acute myeloid leukemia cells. J. Clin. Invest..

[36] Morgenstern J.P., Land H. (1990). Advanced mammalian gene transfer: high titre retroviral vectors with multiple drug selection markers and a complementary helper-free packaging cell line. Nucleic Acids Res..

[37] Naviaux R.K., Costanzi E., Haas M., Verma I.M. (1996). The pCL vector system: rapid production of helper-free, high-titer, recombinant retroviruses. J. Virol..

[38] Palande K.K., Beekman R., van der Meeren L.E., Beverloo H.B., Valk P.J., Touw I.P. (2011). The antioxidant protein peroxiredoxin 4 is epigenetically down regulated in acute promyelocytic leukemia. PLoS ONE.

[39] Jongen-Lavrencic M., Grob T., Hanekamp D., Kavelaars F.G., Al Hinai A., Zeilemaker A., Erpelinck-Verschueren C.A.J., Gradowska P.L., Meijer R., Cloos J. (2018). Molecular Minimal Residual Disease in Acute Myeloid Leukemia. N. Engl. J. Med..

[40] Cong L., Ran F.A., Cox D., Lin S., Barretto R., Habib N., Hsu P.D., Wu X., Jiang W., Marraffini L.A., Zhang F. (2013). Multiplex genome engineering using CRISPR/Cas systems. Science.

[41] Dobin A., Davis C.A., Schlesinger F., Drenkow J., Zaleski C., Jha S., Batut P., Chaisson M., Gingeras T.R. (2013). STAR: ultrafast universal RNA-seq aligner. Bioinformatics.

[42] Trapnell C., Williams B.A., Pertea G., Mortazavi A., Kwan G., van Baren M.J., Salzberg S.L., Wold B.J., Pachter L. (2010). Transcript assembly and quantification by RNA-Seq reveals unannotated transcripts and isoform switching during cell differentiation. Nat. Biotechnol..

[43] Anders S., Pyl P.T., Huber W. (2015). HTSeq--a Python framework to work with high-throughput sequencing data. Bioinformatics.

[44] Love M.I., Huber W., Anders S. (2014). Moderated estimation of fold change and dispersion for RNA-seq data with DESeq2. Genome Biol..

[45] Mootha V.K., Lindgren C.M., Eriksson K.F., Subramanian A., Sihag S., Lehar J., Puigserver P., Carlsson E., Ridderstråle M., Laurila E. (2003). PGC-1alpha-responsive genes involved in oxidative phosphorylation are coordinately downregulated in human diabetes. Nat. Genet..

[46] Subramanian A., Tamayo P., Mootha V.K., Mukherjee S., Ebert B.L., Gillette M.A., Paulovich A., Pomeroy S.L., Golub T.R., Lander E.S., Mesirov J.P. (2005). Gene set enrichment analysis: a knowledge-based approach for interpreting genome-wide expression profiles. Proc. Natl. Acad. Sci. USA.

