# Supplementary material for: Malignant Transformation Involving CXXC4 Mutations Identified in a Leukemic Progression Model of Severe Congenital Neutropenia
Source: Cell Rep Med. 2020 Aug 25;1(5):100074. doi: 10.1016/j.xcrm.2020.100074 (PMC7659587; doi:10.1016/j.xcrm.2020.100074)
Supplement: Document S1. Figures S1–S7 and Tables S1 and S2 [file mmc1.pdf]

Cell Reports Medicine, Volume *1*

Supplemental Information

Malignant Transformation Involving *CXXC4* Mutations Identified in a Leukemic Progression Model of Severe Congenital Neutropenia

Patricia A. Olofsen, Szabolcs Fatrai, Paulina M.H. van Strien, Julia C. Obenauer, Hans W.J. de Looper, Remco M. Hoogenboezem, Claudia A.J. Erpelinck-Verschueren, Michael P.W.M. Vermeulen, Onno Roovers, Torsten Haferlach, Joop H. Jansen, Mehrnaz Ghazvini, Eric M.J. Bindels, Rebekka K. Schneider, Emma M. de Pater, and Ivo P. Touw

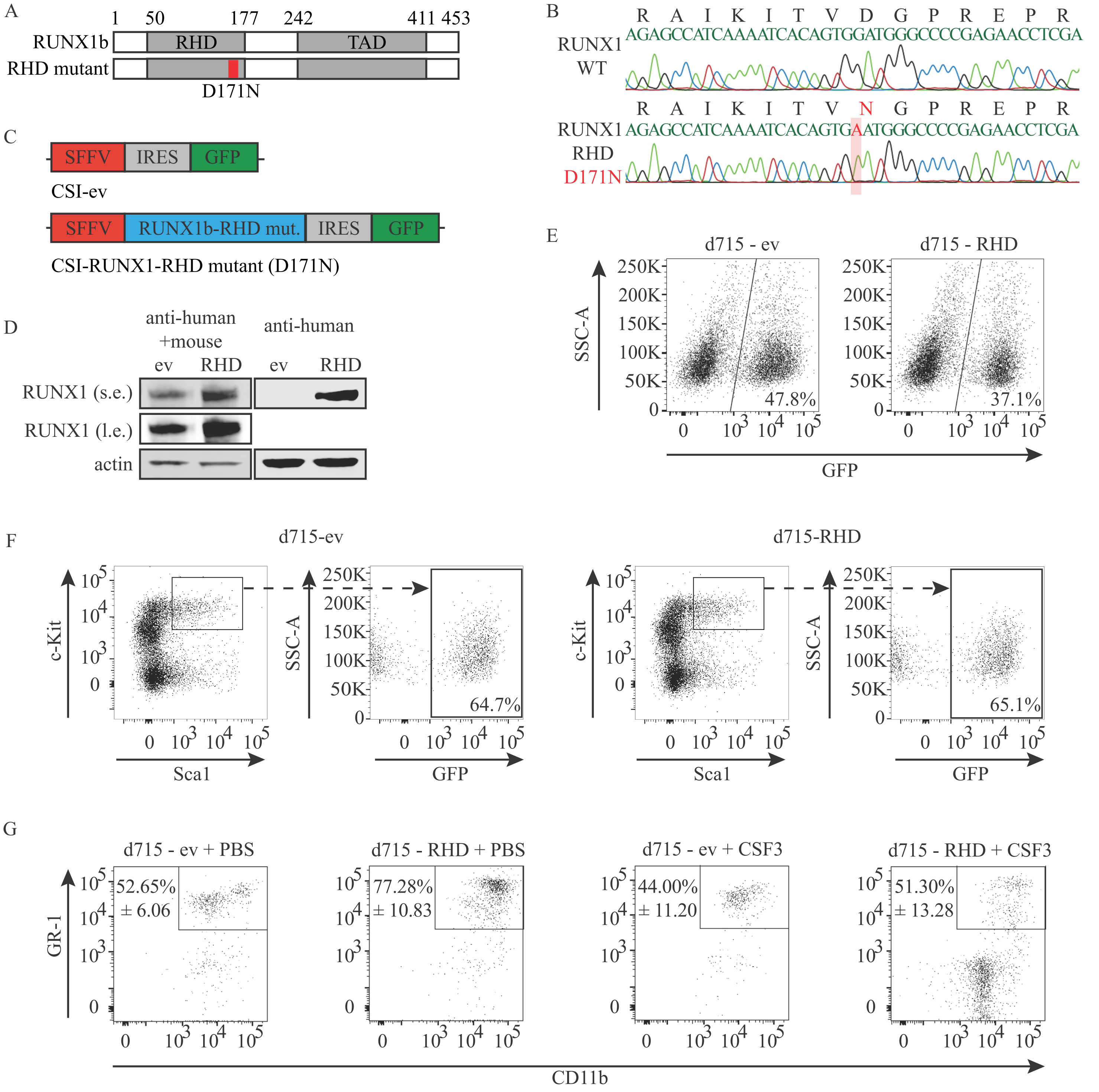

Figure S1: Lentiviral transduction of mouse bone marrow cells (related to Figure 1)
(A,B): Position of the G-to-A missense mutation shown by Sanger sequencing, resulting in D171N amino acid change in RUNX1b (wild type) protein. (C): Lentiviral expression constructs used for transplantation experiments. (D): Expression of mutant RUNX1 protein in lentivirus-transduced lineage depleted hematopoietic progenitors; immunoblot stained with Ab8529 (left panel), detecting both mouse and human RUNX1 after short (s.e.) and long (l.e.) exposure; and with Ab4334 (right panel), specific for human RUNX1. (E): FACS dot plots showing GFP expression in lineage negative cells before transplantation. (F): FACS dot plots showing GFP expression in ~ 65% of LSK cells. (G): Representative FACS dot plots showing GFP+CD11b+GR-1+ neutrophils in the PB. Data are from samples taken 14 and 16 weeks after transplantation (n=11 or 12 per group).

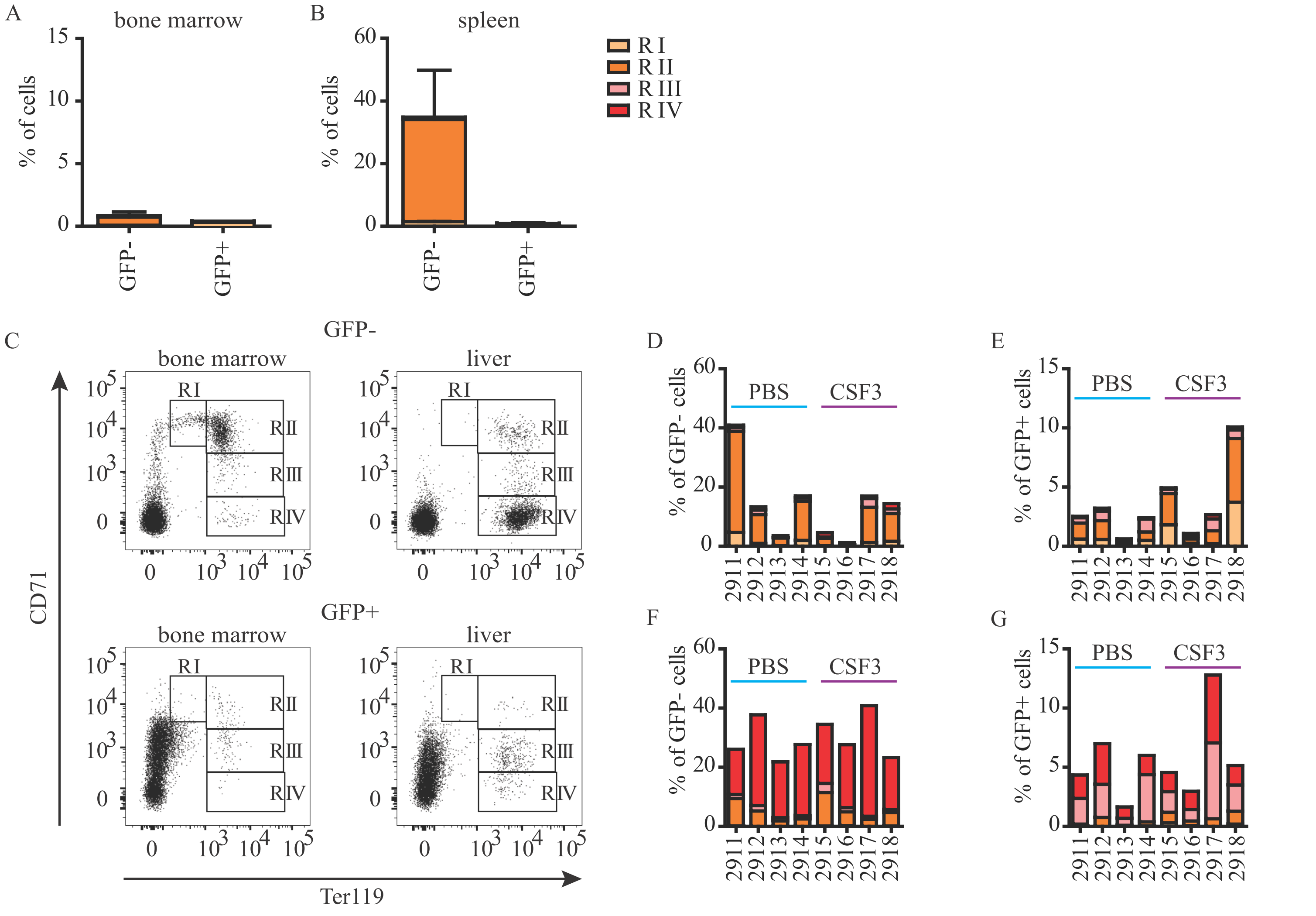

Figure S2: Analysis of erythroid cell populations (related to Figure 2)

(A-G): CD71 and Ter119 staining is used to discriminate between the 4 stages of erythropoiesis with FACS. RI: CD71highTer119-, RII: CD71highTer119+, RIII: CD71intermediateTer119+, RIV: CD71-Ter119+. (A): erythroid cells observed in the bone marrow of the secondary recipients and (B): in the spleen. (C): Representative FACS dot plot showing the different stages of erythroid development based on CD71 and Ter119 in the bone marrow or liver of tertiary recipients. (D): histogram showing erythropoiesis of the GFP- cells in the bone marrow of the tertiary recipients and (E): the GFP+ erythroid cells in the BM. (F): GFP- erythroid cells in the liver and (G): GFP+ erythroid cells in the liver.

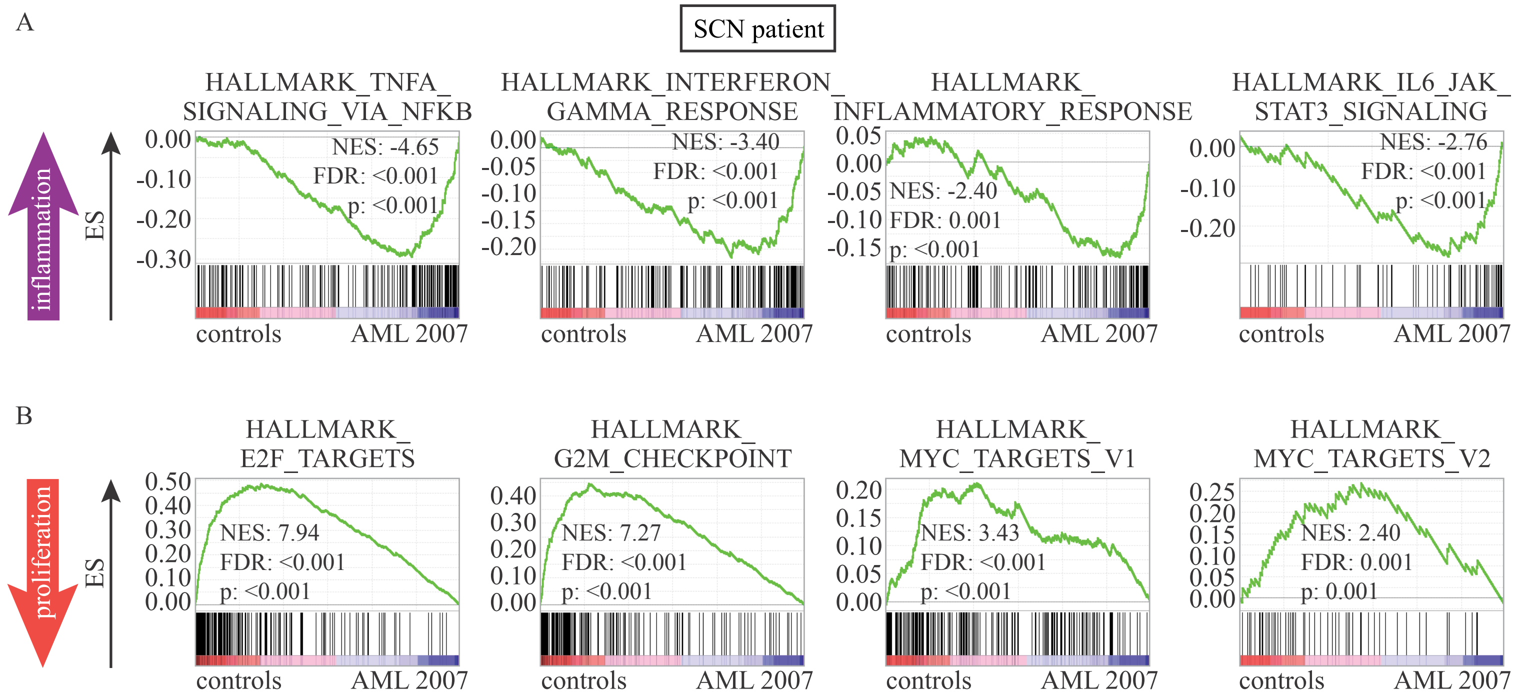

Figure S3: Leukemic progression of SCN (SCN-AML) is associated with increased inflammatory and decreased proliferative signaling (related to Figure 4)

GSEA comparing $CD34^+$ cells from 3 healthy controls with the SCN-AML phase (2007) showing (A): increased inflammatory pathways and (B): down-regulated proliferation signatures. ES = enrichment score, NES = normalized enrichment score, FDR = false discovery rate.

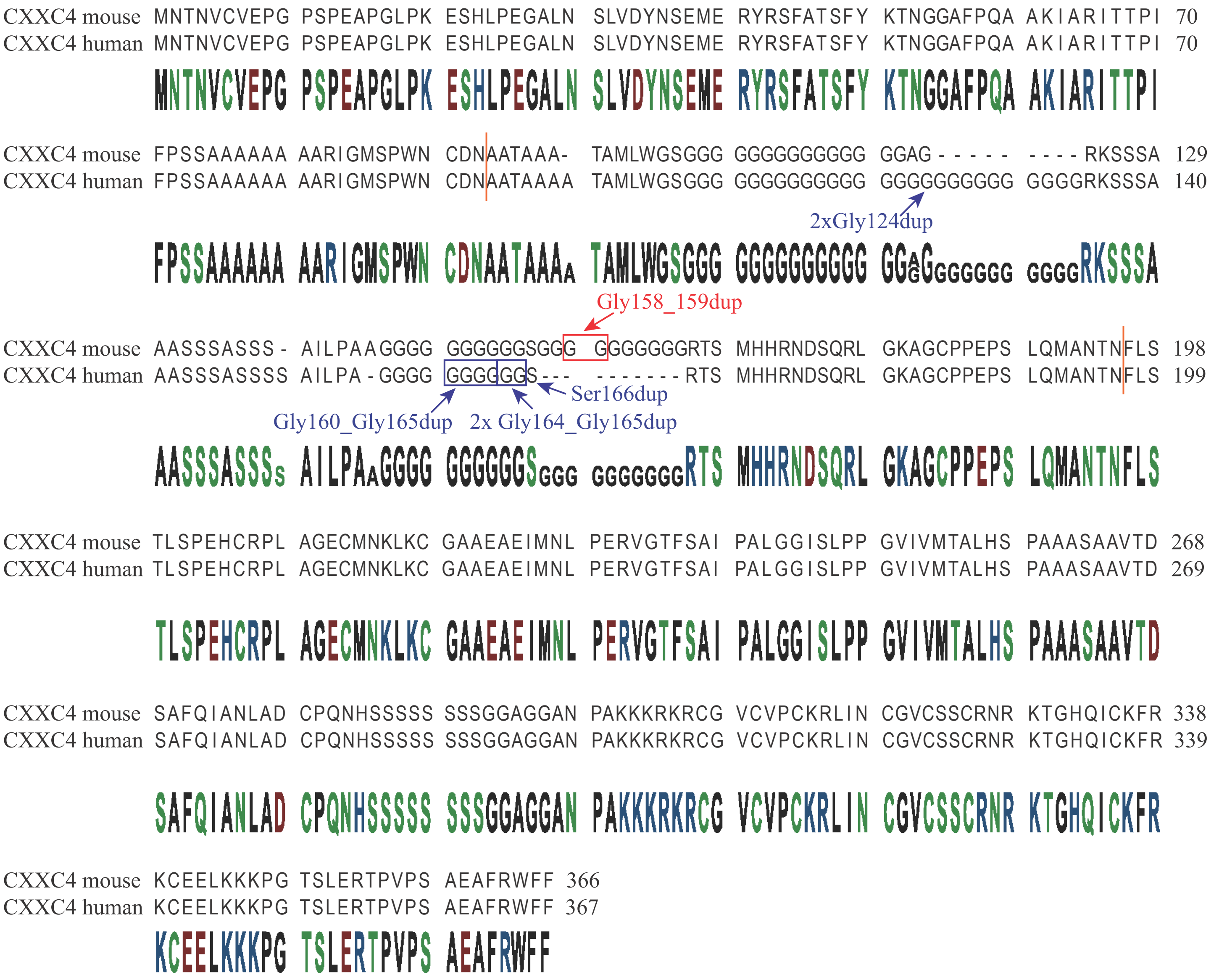

Figure S4: Alignment of mouse and human CXXC4 protein sequences and positioning of mutations in human AML (related to Figures 5 and 6)

Mutations marked in blue indicate alterations found in human AML; the mutation marked in red indicates the CXXC4 alteration found in the murine AML cells. Orange and red bars indicate start and finish of the 2 amplicons used for targeted sequencing.

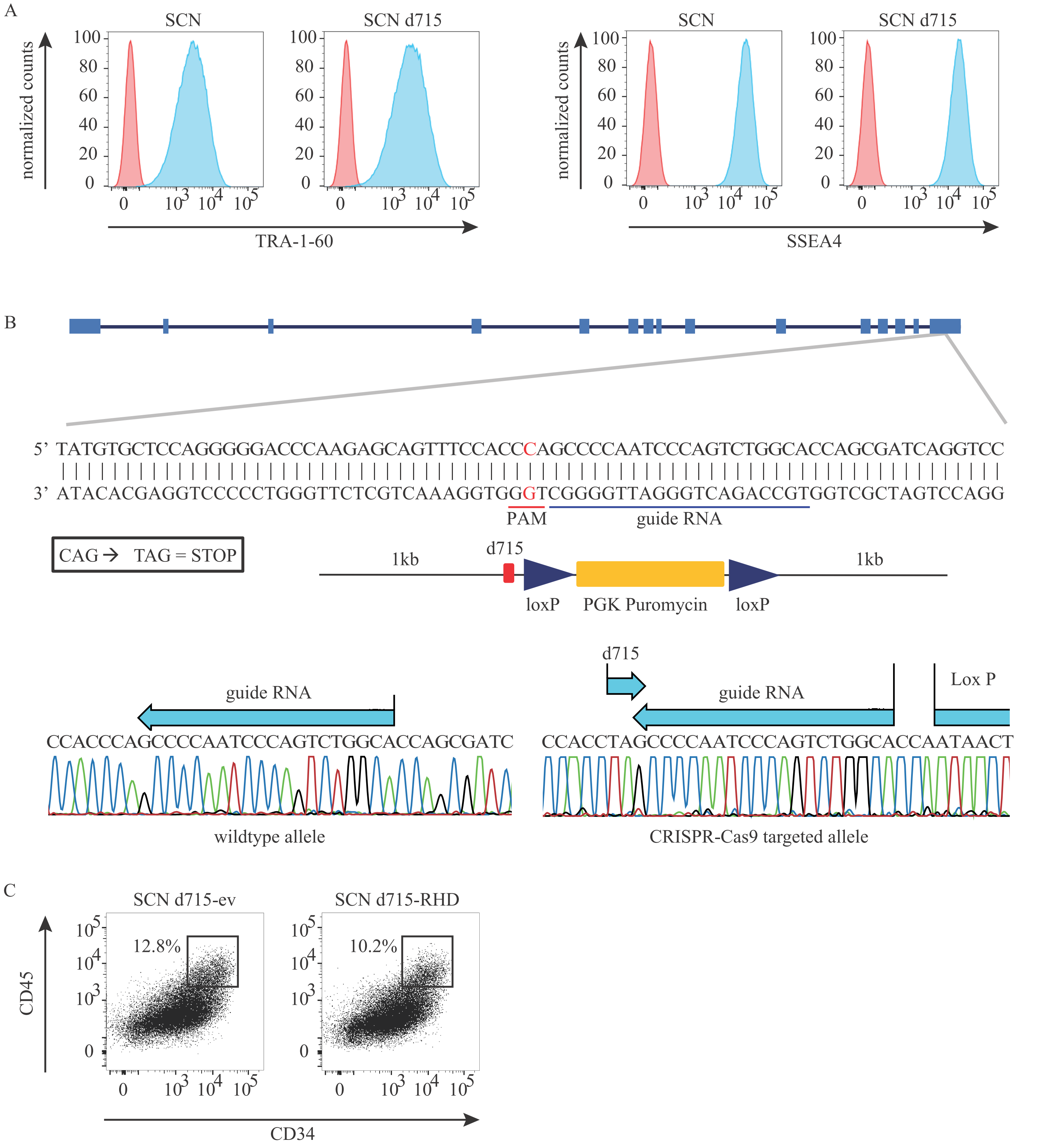

Figure S5: Characterization of pluripotency and the introduction of *CSF3R* mutation in iPSCs (related to Figures 7, S6, S7 and STAR Methods)

(A): FACS histograms (blue) showing TRA-1-60 and SSEA4 expression in SCN-iPSC and SCN-iPSC with the CRISPR-Cas9 targeted *CSF3R*-d715 mutation. Red histograms are from unstained cells. (B): Schematic overview of the recombination template used to introduce the *CSF3R*-d715 mutation with CRISPR-Cas9 and Sanger sequencing data showing the heterozygous integration of the recombination template. (C): FACS dot plot showing CD34 and CD45 expression of floating cells harvest at Day 12 of hematopoietic induction with the STEMdiff Hematopoietic kit.

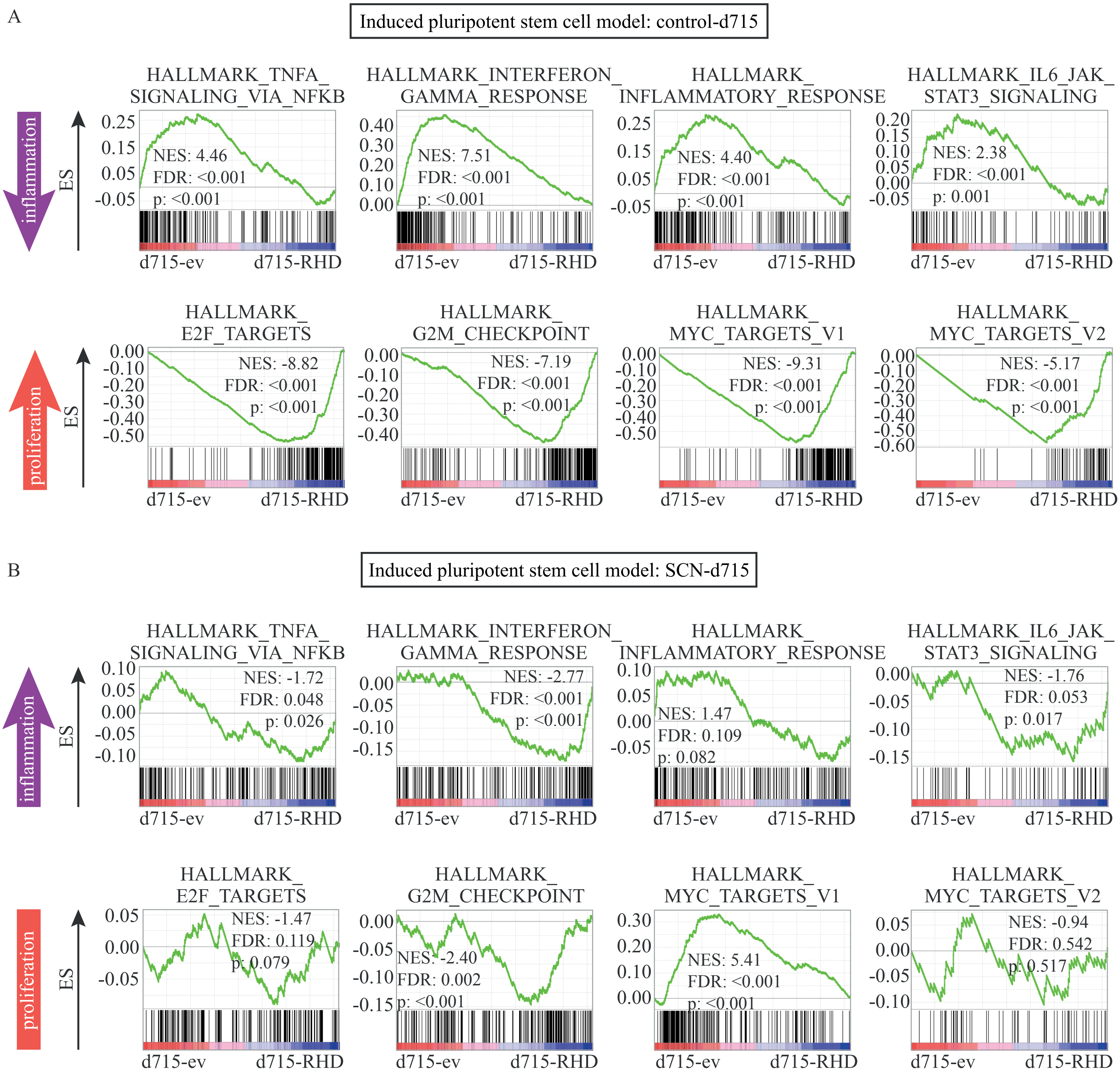

Figure S6: Transcriptome analysis in control and SCN iPSC-derived CD34+CD45+ cells with truncated CSF3R: the SCN background alters the inflammation/ proliferation balance (related to Figures 1, S5)
GSEA analyses comparing CD34$^+$CD45$^+$ cells from *CSF3R*-d715 transduced with either empty vector or RUNX1-RHD in (A): control cells showing reduced inflammatory signaling and increased proliferative signatures, and in (B): *ELANE*-mutant SCN cells showing slightly increased inflammatory signaling, while the proliferation induction is less pronounced. Data shown is derived from 2 independent experiments. ES = enrichment score, NES = normalized enrichment score, FDR = false discovery rate.

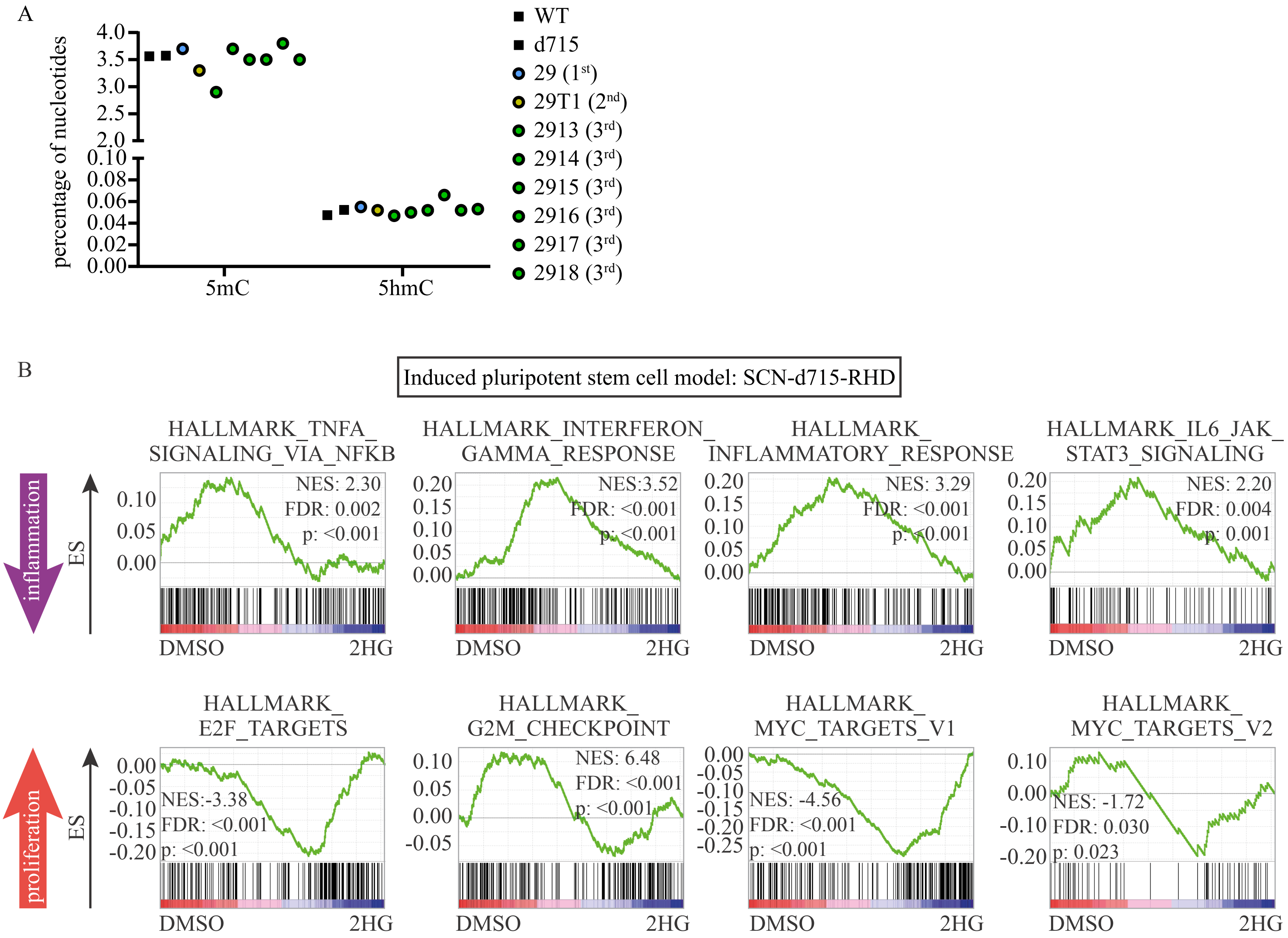

Figure S7: Inhibition of TET2 activity does not mimic the expression signatures observed in AML (related to Figures 4, 7)
(A): Dot plot showing no differences between the percentage of 5mC or 5hmC modified nucleotides in control, primary-, secondary-, and tertiary-transplant recipient mice. Inflammatory and proliferation related GSEA comparing (B): DMSO or 2HG treated, *CSF3R*-d715, *RUNX1*-RHD $CD34^{+}CD45^{+}$ cells derived from ELANE-mutant SCN-iPSC (two independent experiments).

Table S1: Upregulated transcripts in mouse AML and SCN-AML samples (related to Figure 4)

	WT+d715 (TPM)		2911-2918 (TPM)				SCN	SCN-AML	
Transcripts	Mean	SEM	Mean	SEM	p-value	q-value	(TPM)	(TPM)	p-value
ADAP1	**76.610**	7.734	**112.078**	3.714	0.000	0.000	**3.123**	**107.222**	0.007
AKAP12	**1.174**	0.495	**8.178**	0.717	0.000	0.000	**0.125**	**9.114**	0.019
ALDOC	**4.566**	0.518	**20.833**	2.173	0.000	0.000	**4.085**	**66.011**	0.034
ANXA2	**98.767**	2.025	**170.653**	17.325	0.002	0.005	**148.538**	**1166.104**	0.030
ARID3B	**6.817**	0.057	**10.026**	0.784	0.003	0.008	**4.991**	**48.246**	0.040
C2	**0.000**	0.000	**3.416**	0.473	0.000	0.000	**0.000**	**1.029**	0.000
CASS4	**0.989**	0.989	**3.595**	0.339	0.005	0.015	**0.045**	**28.485**	0.002
CDKN1A	**32.308**	4.833	**95.238**	4.043	0.000	0.000	**12.338**	**150.233**	0.022
CISH	**20.392**	0.286	**56.367**	6.222	0.000	0.000	**10.556**	**143.478**	0.022
CSF3R	**81.353**	6.397	**116.220**	6.645	0.000	0.001	**86.063**	**568.637**	0.032
CXCL2	**0.000**	0.000	**1.235**	0.424	0.001	0.004	**92.786**	**1003.455**	0.019
CYTH4	**92.696**	13.180	**142.309**	10.530	0.000	0.000	**8.796**	**127.143**	0.016
DGAT2	**4.360**	0.969	**15.291**	2.522	0.000	0.000	**0.060**	**44.868**	0.001
DUSP1	**74.312**	1.299	**337.066**	110.876	0.018	0.041	**387.031**	**2744.965**	0.035
DUSP2	**129.095**	30.487	**277.417**	46.648	0.019	0.043	**62.289**	**808.883**	0.013
ECHDC2	**0.951**	0.110	**3.173**	0.599	0.014	0.033	**2.398**	**59.611**	0.023
EFNA1	**2.406**	1.089	**8.496**	1.225	0.000	0.001	**2.614**	**66.791**	0.020
ESAM	**37.701**	1.076	**111.813**	12.971	0.000	0.000	**17.079**	**129.748**	0.048
F13A1	**47.495**	8.898	**510.433**	86.608	0.000	0.000	**17.280**	**705.205**	0.003
FAM49A	**10.927**	0.263	**14.803**	0.993	0.004	0.012	**0.093**	**52.449**	0.000
FGD5	**2.089**	0.546	**14.054**	1.059	0.000	0.000	**0.595**	**26.595**	0.008
FOS	**41.011**	16.634	**457.660**	181.649	0.004	0.011	**436.335**	**6789.652**	0.010
GDPD5	**0.224**	0.224	**4.420**	0.626	0.000	0.000	**0.000**	**61.903**	0.000
GNG11	**32.207**	4.152	**43.629**	2.385	0.004	0.011	**51.361**	**489.900**	0.028
HCK	**0.672**	0.518	**32.154**	3.692	0.000	0.000	**19.422**	**341.296**	0.012
HIP1	**2.020**	0.294	**14.266**	0.459	0.000	0.000	**2.172**	**19.226**	0.047
ICAM1	**44.452**	10.317	**84.731**	3.274	0.000	0.000	**14.345**	**154.835**	0.027
IER2	**273.214**	19.229	**847.096**	137.990	0.000	0.002	**344.232**	**3173.270**	0.023
IL10RA	**9.567**	5.397	**23.257**	3.637	0.012	0.030	**22.399**	**275.262**	0.018
KCNK6	**2.574**	1.785	**9.410**	0.902	0.000	0.000	**0.965**	**37.293**	0.009
KLF2	**2.028**	0.239	**19.443**	9.241	0.007	0.017	**14.971**	**128.116**	0.036
MAP3K1	**1.557**	0.415	**4.109**	0.262	0.000	0.000	**0.352**	**11.187**	0.028
MIR22HG	**0.000**	0.000	**3.166**	0.754	0.000	0.000	**4.310**	**11.151**	0.040
MYRF	**0.000**	0.000	**1.503**	0.311	0.000	0.000	**0.000**	**17.450**	0.001
PARP8	**29.957**	11.253	**50.302**	3.074	0.000	0.002	**1.413**	**25.464**	0.022
PDE2A	**17.286**	1.015	**40.984**	4.261	0.000	0.000	**0.051**	**63.809**	0.000
PEAK1	**0.876**	0.174	**4.056**	0.247	0.000	0.000	**0.172**	**11.633**	0.011
PELI2	**4.112**	0.100	**5.844**	0.292	0.002	0.007	**1.817**	**27.706**	0.028
PLK2	**1.958**	0.831	**4.445**	0.598	0.011	0.027	**0.000**	**37.952**	0.001
PMAIP1	**1.514**	0.463	**181.861**	9.115	0.000	0.000	**112.887**	**667.623**	0.047
POU2F2	**5.479**	0.488	**10.329**	0.980	0.001	0.003	**0.894**	**14.981**	0.047
PPAP2A	**4.018**	2.135	**7.460**	0.526	0.013	0.030	**0.040**	**109.365**	0.001
PTGIR	**2.619**	0.815	**9.546**	0.698	0.000	0.000	**14.987**	**193.512**	0.020
RBPMS	**73.450**	12.444	**159.384**	11.375	0.000	0.000	**22.089**	**243.656**	0.018
RHOB	**7.714**	0.559	**24.352**	4.253	0.000	0.000	**7.875**	**75.929**	0.044
RXFP1	**0.000**	0.000	**2.603**	0.242	0.000	0.000	**2.255**	**218.103**	0.002
S100A16	**0.101**	0.101	**15.640**	3.291	0.000	0.000	**0.450**	**201.869**	0.000
SGCE	**15.925**	0.527	**41.888**	2.877	0.000	0.000	**0.848**	**81.740**	0.012
SORT1	**17.619**	1.869	**31.723**	1.231	0.000	0.000	**1.734**	**49.372**	0.008
SPARC	**1.000**	0.918	**15.064**	1.596	0.000	0.000	**137.496**	**772.904**	0.049
SPP1	**5.969**	2.560	**98.210**	6.977	0.000	0.000	**4.538**	**98.258**	0.022
TNNT1	**2.163**	0.091	**37.240**	2.072	0.000	0.000	**0.000**	**63.938**	0.001
VNN1	**1.068**	1.068	**5.946**	0.614	0.000	0.000	**19.620**	**148.520**	0.039
YES1	**0.469**	0.263	**1.798**	0.292	0.002	0.006	**1.096**	**70.475**	0.003
ZFP36	**62.796**	9.864	**341.485**	84.642	0.001	0.003	**372.547**	**2042.670**	0.049

Table S2: Downregulated transcripts in mouse AML and SCN-AML samples (related to Figure 4)

	WT+d715 (TPM)		2911-2918 (TPM)				SCN (TPM)	SCN-AML (TPM)	
Transcripts	Mean	SEM	Mean	SEM	p-value	q-value			p-value
ABLIM1	**3.135**	2.449	**0.443**	0.180	0.003	0.008	**17.242**	**0.000**	0.000
ALDH1A1	**40.810**	18.173	**2.811**	0.750	0.000	0.000	**267.785**	**1.687**	0.001
APOE	**1457.806**	75.156	**139.495**	26.103	0.000	0.000	**151.051**	**10.498**	0.039
BLNK	**8.679**	2.850	**0.087**	0.041	0.000	0.000	**71.718**	**5.069**	0.042
BLVRB	**124.042**	8.566	**34.675**	6.532	0.000	0.000	**348.949**	**30.415**	0.035
CEP152	**21.006**	1.614	**10.996**	0.601	0.000	0.000	**46.303**	**3.988**	0.038
CHST2	**0.732**	0.250	**0.116**	0.036	0.010	0.024	**45.965**	**0.026**	0.000
CKAP2L	**158.325**	12.128	**69.921**	3.530	0.000	0.000	**38.703**	**3.558**	0.047
DEPDC1B	**90.213**	13.161	**41.690**	2.524	0.000	0.000	**127.093**	**12.717**	0.041
DHX32	**47.015**	10.050	**14.669**	1.364	0.000	0.000	**72.522**	**4.681**	0.034
DTL	**150.428**	0.056	**59.978**	2.771	0.000	0.000	**109.663**	**14.026**	0.049
DYNLT3	**133.093**	10.515	**63.246**	2.294	0.000	0.000	**50.740**	**0.000**	0.001
EPOR	**103.405**	2.778	**23.938**	5.811	0.001	0.004	**100.145**	**1.177**	0.005
FAM178B	**1.034**	0.402	**0.000**	0.000	0.000	0.001	**183.409**	**0.364**	0.002
FRMD6	**14.164**	2.754	**8.098**	0.623	0.009	0.023	**25.186**	**0.000**	0.001
FZD3	**1.105**	0.460	**0.190**	0.027	0.000	0.000	**6.719**	**0.002**	0.002
GATA1	**370.954**	32.021	**48.081**	15.706	0.003	0.008	**427.197**	**4.972**	0.002
GNG2	**32.650**	0.353	**10.757**	1.946	0.000	0.001	**65.696**	**4.499**	0.027
GPR183	**6.159**	1.352	**0.850**	0.260	0.000	0.000	**74.745**	**4.742**	0.042
HMMR	**71.704**	8.790	**42.926**	3.632	0.005	0.014	**118.365**	**4.299**	0.015
HNRNPLL	**178.291**	2.327	**19.761**	4.419	0.000	0.000	**48.831**	**0.697**	0.005
IGLL1	**18.724**	1.757	**0.063**	0.063	0.000	0.000	**937.323**	**55.152**	0.015
IL1B	**3.883**	0.750	**0.142**	0.079	0.000	0.002	**263.481**	**27.556**	0.040
KCNH2	**1.367**	0.451	**0.268**	0.098	0.022	0.048	**219.696**	**0.744**	0.001
KIF15	**43.094**	0.878	**26.427**	1.275	0.000	0.000	**52.337**	**3.100**	0.024
KLF1	**454.940**	39.636	**52.026**	20.291	0.006	0.015	**700.309**	**4.974**	0.001
LEF1	**3.373**	1.681	**0.032**	0.024	0.000	0.000	**66.537**	**0.022**	0.001
LTBP1	**4.494**	1.367	**0.084**	0.024	0.000	0.000	**41.812**	**2.023**	0.018
MMRN1	**35.972**	0.586	**6.478**	1.504	0.000	0.001	**55.165**	**2.381**	0.015
MS4A2	**49.546**	3.729	**8.008**	3.627	0.000	0.000	**34.475**	**1.267**	0.023
MYCT1	**38.009**	6.315	**23.830**	1.273	0.006	0.015	**28.496**	**0.000**	0.001
NDN	**12.449**	2.148	**0.119**	0.063	0.000	0.000	**52.644**	**1.734**	0.023
NHLRC1	**17.069**	1.609	**9.799**	0.890	0.005	0.014	**67.709**	**2.740**	0.021
NMNAT3	**56.707**	0.478	**29.187**	1.108	0.000	0.000	**126.918**	**3.366**	0.007
NUDT12	**22.107**	1.271	**6.863**	0.514	0.000	0.000	**23.473**	**1.334**	0.046
OAT	**314.230**	18.419	**178.851**	6.536	0.000	0.000	**290.159**	**40.190**	0.046
PDZD8	**26.334**	1.826	**17.264**	1.248	0.004	0.011	**62.318**	**5.040**	0.026
PTPN22	**46.281**	8.276	**12.565**	1.787	0.000	0.000	**43.349**	**1.812**	0.025
REC8	**3.687**	0.059	**1.097**	0.234	0.007	0.018	**291.910**	**3.809**	0.003
SLAIN1	**65.971**	2.351	**32.809**	3.312	0.000	0.001	**61.145**	**0.036**	0.000
SLC14A1	**298.487**	11.272	**10.699**	3.687	0.000	0.000	**22.432**	**1.285**	0.046
SLC18A2	**122.849**	1.763	**28.599**	5.559	0.001	0.002	**20.948**	**0.013**	0.018
SLC44A1	**9.040**	1.376	**0.812**	0.230	0.000	0.000	**28.705**	**2.720**	0.039
SNX9	**106.569**	2.147	**37.675**	1.870	0.000	0.000	**35.243**	**1.191**	0.015
STEAP3	**40.553**	1.689	**13.375**	1.970	0.000	0.000	**40.457**	**3.494**	0.047
TGM2	**19.927**	6.583	**3.418**	0.704	0.000	0.000	**59.561**	**1.928**	0.011
TMEM246	**14.893**	0.026	**0.842**	0.120	0.000	0.000	**64.100**	**0.051**	0.000
TOP2A	**187.231**	10.462	**75.674**	4.452	0.000	0.000	**117.467**	**15.956**	0.049
TRIB2	**236.288**	7.422	**11.866**	4.045	0.000	0.001	**84.766**	**4.264**	0.015
TSPYL5	**0.766**	0.766	**0.000**	0.000	0.022	0.049	**32.012**	**0.000**	0.000
TTK	**54.824**	12.176	**31.376**	2.024	0.003	0.010	**52.528**	**3.994**	0.038
WEE1	**137.495**	0.252	**59.770**	1.893	0.000	0.000	**183.638**	**22.740**	0.045
XK	**30.586**	1.902	**14.624**	1.372	0.000	0.000	**32.481**	**0.042**	0.001
